# Natural history in Malan syndrome: survey of 28 adults and literature review

**DOI:** 10.1186/s13023-024-03288-6

**Published:** 2024-07-29

**Authors:** T. N. Huynh, C. G. Delagrammatikas, L. Chiriatti, A. Panfili, K. Ventarola, L. A. Menke, M. Tartaglia, S. A. Huisman, M. Priolo

**Affiliations:** 1 Department of Pediatrics, Emma Children’s Hospital, Amsterdam UMC, University of Amsterdam, Meibergdreef 9, 1105 AZ Amsterdam, The Netherlands; 2 Director of Research, Malan Syndrome Foundation, Old Bridge, NJ USA; 3 Molecular Genetics and Functional Genomics, Ospedale Pediatrico Bambino Gesù, IRCCS, Viale di San Paolo 15, 00146 Rome, Italy; 4 Scientific Directorate, Fondazione Policlinico Universitario A. Gemelli IRCCS, Rome, Italy; 5 Medical Genetics Unit, Fondazione Policlinico Universitario A. Gemelli IRCCS, Rome, Italy; 6 Malan Syndrome Foundation, Old Bridge, NJ USA; 7 Department of Pediatrics, Emma Children’s Hospital, Amsterdam UMC, University of Amsterdam, Amsterdam Reproduction and Development Research Institute, Meibergdreef 9, 1105 AZ Amsterdam, The Netherlands; 8 Zodiak, Prinsenstichting, 1444 JE Purmerend, The Netherlands; 9 Operative Unit of Medical Genetics and Laboratory of Genetics, AORN A.Cardarelli, Via Cardarelli 9, 80131 Naples, Italy

**Keywords:** Malan syndrome, Natural history, Adult phenotype, Overgrowth syndrome, Sotos syndrome, Intellectual disability, Management recommendations, Rare diseases

## Abstract

**Background:**

Malan syndrome (MALNS), previously referred to as “Sotos syndrome 2” due to its resemblance to Sotos syndrome (SS), is an ultra-rare neurodevelopmental disorder characterized by overgrowth, typical craniofacial features, intellectual disability (ID), and a range of psychobehavioral, musculoskeletal, vision and neurological signs. As MALNS and SS partly overlap, it is essential to more accurately profile their clinical presentations and highlight their differences in order to improve syndrome specific management. An increasing number of individuals with MALNS reach adult-age though the natural history of the disorder is poorly characterized due to the small number of adult individuals described so far. As a consequence, current guidelines are limited to the pediatric population. Further delineation of MALNS is essential to optimize care in adulthood.

**Results:**

A mixed approach based on cross-sectional data collection with a survey disseminated to caregivers of adults with molecularly confirmed MALNS and literature review was conducted. Twenty-eight caregivers completed the survey. Clinical presentation in adulthood is multisystemic and defined by psychobehavioral comorbidities (96%), musculoskeletal involvement (96%), vision impairment (96%) and neurological complications (86%). The most common signs were anxiety (79%), hypotonia (75%), movement difficulty (75%), scoliosis (64%), problems with coordination (61%), strabismus (57%), constipation (54%), breastbone abnormalities (54%) and advanced bone age during childhood (54%). Impaired vision was complicated by vision decline (36%) and optic atrophy (32%). We report some previously unidentified features, including high pain threshold (46%), incontinence (25%), tremors (21%), muscle hypoplasia (18%) and tics (18%).

**Conclusions:**

This survey in the adult population has allowed a more complete description of the natural history of MALNS. Our findings will contribute to the development and improvement of standards of care for adults with MALNS to assure optimal health monitoring and treatment of evolutive complications. We propose additional recommendations to the previous dataset of clinical evaluations specifically applied to adults. The comparison of MALNS and SS adult presentation highlights significant differences in terms of prevalence and severity of ID, behavioral issues, and vision problems, confirming that a proper differential diagnosis between the two conditions is indispensable to guide physicians and mental health professionals to syndrome specific management.

**Supplementary Information:**

The online version contains supplementary material available at 10.1186/s13023-024-03288-6.

## Background

“Doctor, as our child with intellectual disability (ID) gets older and becomes an adult, what can we expect with such a rare condition?” This is one of the major questions that affected individuals and their caregivers raise to their medical providers in an era where technological advances and diagnostic testing are leading to the identification of an increasing number of previously unrecognized rare and ultra-rare disorders [1, 2]. Individuals with developmental delay (DD)/ID are generally molecularly diagnosed at a young age simply because they are under routine pediatric care which increases their chances of receiving proper genetic testing. Similarly, research studies devoted to disclosing genetic causes in rare disorders are mainly conducted in the pediatric population. Consequently, most of the available clinical information concerns childhood; this limitation does not expand on the full spectrum of the disorder and its phenotypical changes over time, also known as the natural history [3].

Malan syndrome (MALNS) (MIM #614753) is an ultra-rare genetic disorder with an estimated prevalence of < 1/1,000,000 [4]. It is caused by haploinsufficiency of the nuclear factor I X gene (*NFIX*, MIM #164005), due to either heterozygous chromosomal microdeletions involving the 19p13.2 region or loss-of-function (LoF) variants in the *NFIX* gene, these latter mostly located within exons 2 to 4 [5, 6]. NFIX plays an important role in cellular processes during brain and musculoskeletal development. Since its first description in 2010, MALNS has been referred to as “Sotos syndrome 2” or “Sotos-like” as it presents with some similarities to Sotos syndrome (SS, MIM #117550) [6]. Main characteristics of MALNS include overgrowth at early age, macrocephaly, distinct craniofacial features, DD/ID, and neurological, psychobehavioral, ophthalmological, and musculoskeletal features [5]. Presently, less than 100 individuals with MALNS have been reported worldwide [5, 7]. While it is likely that an increasing number of affected individuals will reach adult-age, literature on the manifestations in adulthood remains limited. Priolo et al. have provided an overview of clinical phenotypes in a large cohort of individuals with MALNS. A total of 80 subjects with ages ranging from 1 to 42 years old have been described. Of these, only thirteen individuals (16%) were of adult-age at the time of assessment [5]. Moreover, this study did not report data on daily functioning, medical and psychobehavioral management, and impact on adult quality of life (QoL).

Currently, limited guidelines mainly related to the pediatric population of MALNS exist to help with management and follow-up, consequently the support is not always optimal for all ages [7]. Detailed information is essential for optimal care in later life. Adult individuals with moderate to severe ID are often not able to provide reliable information about their condition. Instead, many rely on their caregivers, who can provide valuable information on their health and behavior. Patient advocacy organizations and support groups represent a significant resource for collecting and sharing data on rare population cohorts [3, 8].

In collaboration with the international patient advocacy group, the Malan Syndrome Foundation, this study characterizes the natural history of MALNS in adult individuals as reported by their caregivers. Given the range of comorbidities in MALNS, the participants were asked to answer the following primary question: which medical and psychobehavioral problems are still present in adulthood? Secondly, we examined the age of onset to establish which problems manifested at adult age. Other questions of the study were mainly represented by daily functioning, the impact of medical problems on adult QoL, the medical and psychobehavioral treatments and their outcomes. The data collected on adult MALNS individuals in this study were then compared to adult manifestations in SS.

## Methods

A mixed descriptive study design was used, including a literature review and cross-sectional caregiver-reported data collection with an online survey.

### Literature review

PubMed and EMBASE databases were searched to find publications using key words “Malan syndrome” OR “MALNS” OR “Malan overgrowth” OR “Sotos type 2” OR “Sotos type II” OR “NFIX overgrowth” OR “Sotos syndrome 2” OR “Sotos-like”. Only publications in English or Dutch language were included. Duplications were removed manually. Articles were excluded if they did not describe adult individuals with MALNS. Citations of included articles were reviewed to identify additional relevant publications. A single article, reporting a detailed clinical characterization of a large cohort of adult individuals with SS was used for comparison to current data. [9].

### Survey

#### Study population

Subjects eligible to participate in this study were parents or primary caregivers of adult individuals (18 years and older) with a molecularly confirmed diagnosis of MALNS. Participants were internationally recruited in collaboration with the Malan Syndrome Foundation and Malan syndrome centers of expertise in AORN “A. Cardarelli” in Naples and Amsterdam University Medical Centers. Participation was voluntary.

#### Procedure

The English language-survey was adapted from two dedicated surveys: the adult natural history Rubinstein-Taybi syndrome study (Douzgou et al. [10]) and Sanford CoRDS Patient Registry [11] and modified with disease- and adult-specific questions for MALNS. The survey consisted of 115 questions and included closed (single or multiple choice) and open-ended questions. They comprised thirteen themes: general information, neurological, musculoskeletal, cardiovascular, gastrointestinal, respiratory, sleep, vision, and hearing, psychobehavioral, other medical problems, everyday life, medical and psychobehavioral treatments, and follow-up. Additionally, respondents were asked if current or past medical problems had an impact on adult QoL and to specify the medical problem if this was the case (see Additional file 1).

All participants received an email sent through Castor EDC, a secured electronic clinical data management platform, which contained a link to the online survey between April 2023 and May 2023. Participants with limited English proficiency received assistance from their physician or members of the research team to fill out the survey. Researchers were permitted to enter participant’s responses in Castor EDC if the participants needed assistance. Reminders were sent out through the Malan Syndrome Foundation.

#### Data management

Data was pseudo-anonymized and stored in Castor EDC. Participants were coded by serial number, and their email was linked for survey dissemination. IP-addresses were not collected. Access to the database was only granted to members of the research team.

### Statistical analyses

Data was analyzed using IBM SPSS Statistics (version 28) and descriptive statistics were performed to calculate frequencies and percentages. The Fisher’s exact test was used to compare symptoms between MALNS and SS, and differences with a *p* value < 0.05 were considered statistically significant.

## Results

### Literature review

Literature search yielded eight articles with nineteen molecularly confirmed MALNS adult individuals (10 men and 9 women; age range: from 18 to 42 years, median 22 years). [5–7, 12–16] Full descriptions per adult individual are available in Supplementary Table 1 (see Additional file 2). The most frequent reported phenotypic features are displayed in Supplementary Fig. 1 (see Additional file 3) in comparison to results of this survey. Nearly everyone had manifestations of overgrowth: postnatal height > 2 SD (11/18) and head circumference > 2 SD (14/17). Typical MALNS craniofacial features were present in all individuals, mainly represented by prominent forehead (19/19) and chin (16/19) with a long, narrow, and triangular face (18/18). Impaired vision was mostly attributed to strabismus (10/18) and refractive disorders (8/16). Psychobehavioral comorbidities had a high prevalence: autistiform traits (8/18) and anxiety (4/6) were often observed. Priolo et al. [5] reported anxiety in 52% of cases, including children and adults, but stratified prevalence in childhood and adulthood was not provided.

### Survey

#### Demographic data

**Fig. 1:**
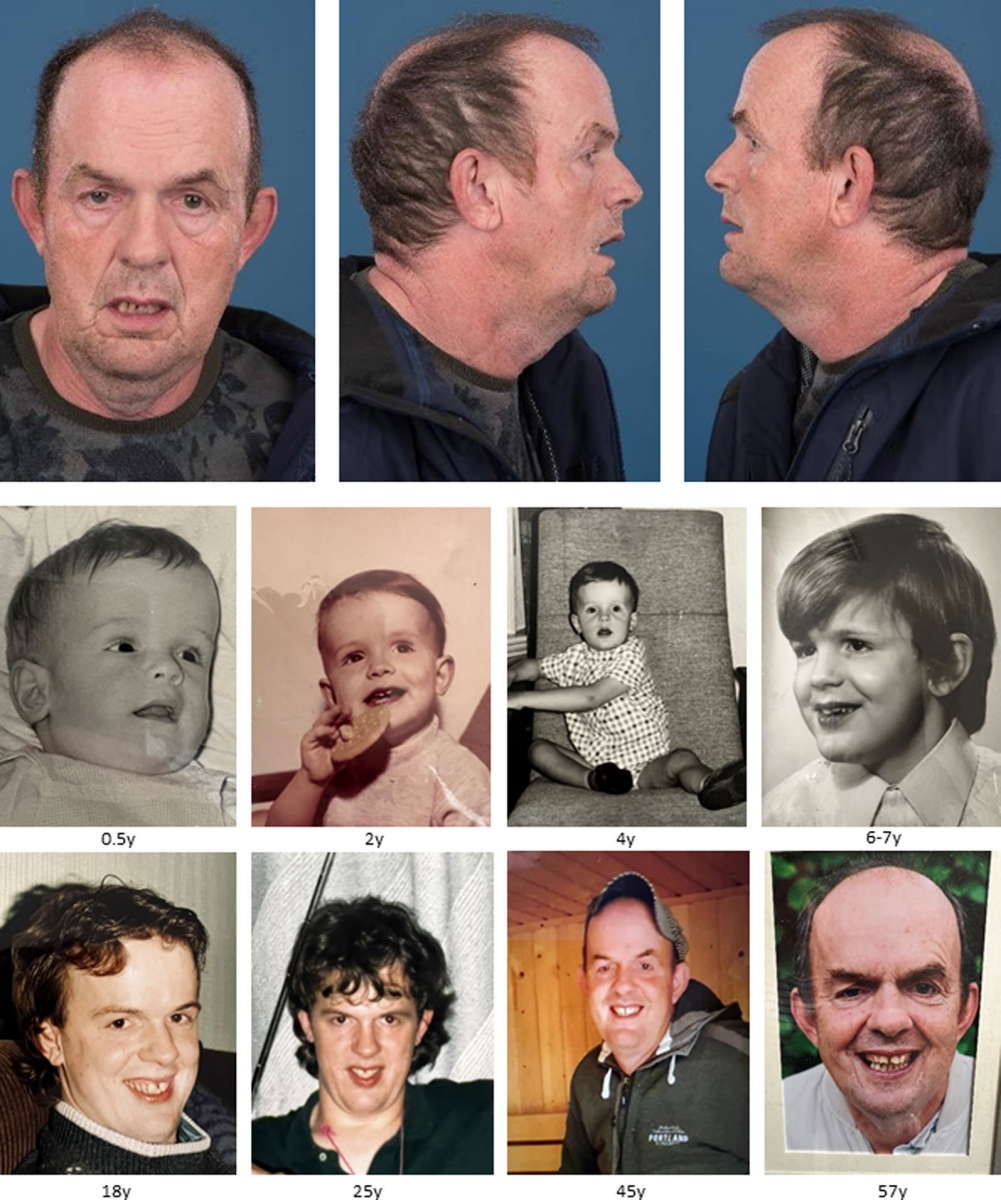
Phenotypical appearance of the oldest participant of the present survey (60 years old) and his age-related changes. Age in years is described below each picture

A total of 28 respondents (16 men and 12 women; age range: 18–60 years (median 23.5 years)) completed the survey from eight different countries: Australia, Canada, Chile, Hungary, Italy, the Netherlands, United Kingdom, and United States of America. All respondents were first-degree family members of an adult individual with molecularly confirmed MALNS. Age distribution is shown in Supplementary Fig. 2 (see Additional file 3). Phenotypical appearance of the oldest individual (60 years) and age-related changes are shown in Fig. 1. Among the participants, seven have been previously described as children in Priolo et al. 2012 (subject 20) and 2018 (subjects 8 and 28), Gurrieri et al. (subject 18) and Macchiaiolo et al. (subjects 19, 21 and 22) [5, 7, 17, 18]. These individuals were contacted to ask for their participation in this adult survey.

Most of the individuals (24/28; 86%) lived at home with their primary caregiver(s), while the remaining (4/28; 14%) lived in assisted living homes. Except for one, all individuals were molecularly confirmed at the age of 10 years or older. Further detailed genotypical data is illustrated in Supplementary Table 2 (see Additional file 3). Microdeletions were present in six individuals (21%).

The male mean height, weight, and head circumference was 185.7 cm (+ 1.29 SD), 75.1 kg (+ 0.37 SD), and 60.9 cm (+ 4 SD), respectively, compared to women’s 177.3 cm (+ 2.18 SD), 64.1 kg (+ 0.41 SD), and 61.0 cm (+ 6 SD), respectively.

#### Daily functioning

**Fig. 2:**
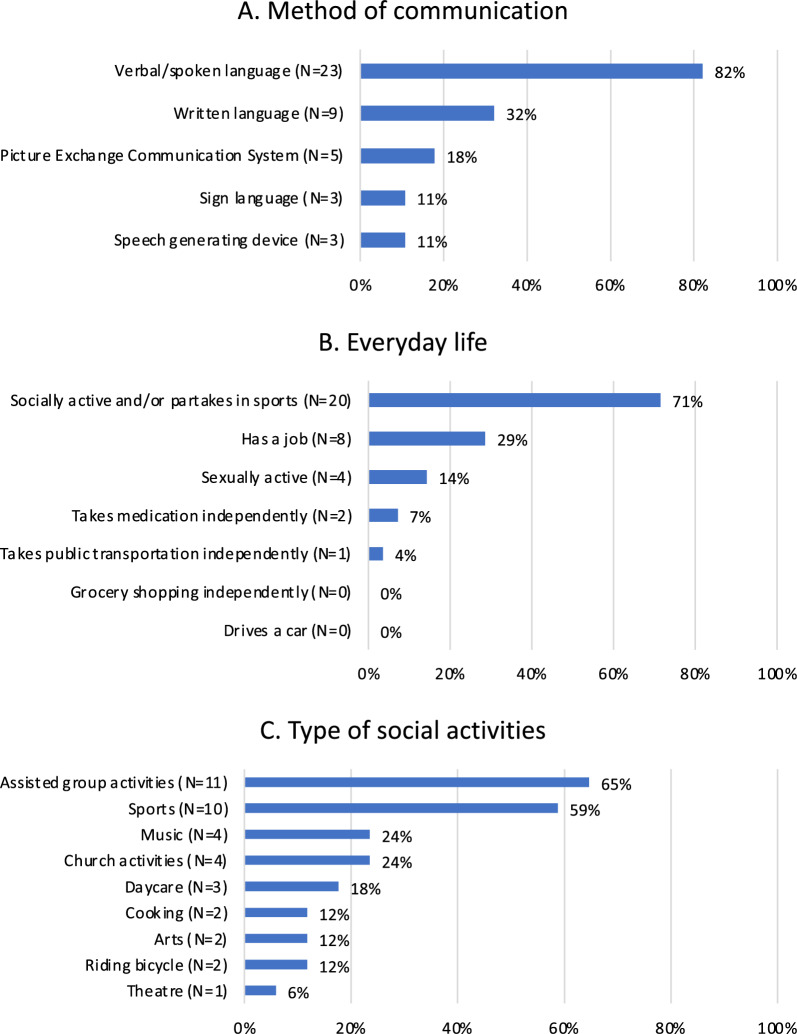
Daily functioning. **A**. Method of communication **B**. Everyday life **C**. Type of social activities

Among the individuals who communicated verbally (82%), 22 caregivers provided details about their child’s level of speech: 16/22 (73%) had a level of speech using 5 + word sentences, 4/22 (18%) communicated with 2–3-word combinations, and 2/22 (9%) communicated with single words. For those with limited verbal communication, sign language and/or alternative/augmentative communication systems were used (Fig. 2A). Twenty-one caregivers reported on changes from childhood to adulthood. Among them, 16 (76%) experienced an improvement in speech, four (19%) observed no changes, and one caregiver stated a gradual decline in level of speech in their affected 43-year-old son. Descriptions of daily functioning including method of communication, level of independency in everyday life, and type of social activities is shown in Fig. 2A, B, and C respectively. The majority of individuals were socially active (Fig. 2B), participating in a variety of activities (Fig. 2C). Eight individuals (29%) had a job with supervision. However, none of the individuals was independent in their everyday life (e.g., ability to go grocery shopping independently). Only one individual (3.6%) was able to take public transportation without assistance.

#### Health and behavior in adulthood

**Fig. 3:**
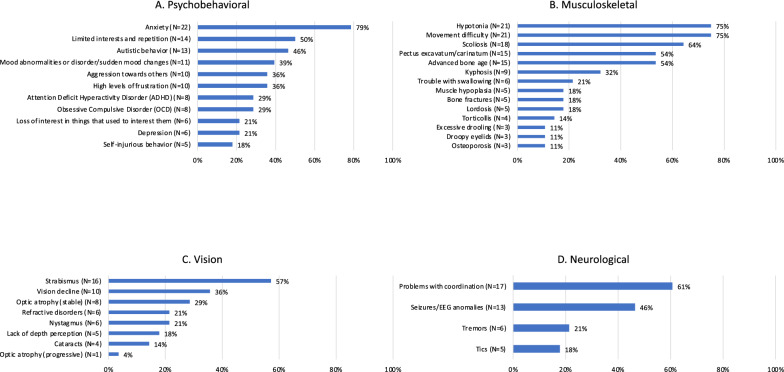
Features per body system. **A**, Psychobehavioral; **B**, Musculoskeletal; **C**, Vision;** D**, Neurological

All caregivers reported multisystemic involvement. The most frequent issues were represented by psychobehavioral comorbidities (27/28; 96%), musculoskeletal involvement (27/28; 96%) and vision impairment (27/28; 96%). Neurological complications (24/28; 86%) were also common. Detailed information on signs and symptoms of the aforementioned four body systems is illustrated in Fig. 3. When considering psychobehavioral problems, anxiety was observed in the majority of individuals (22/28; 79%). Other frequently reported psychobehavioral problems included limited interests, repetitive movements and echolalia (14/28; 50%), autistic behavior (13/28; 46%), and mood abnormalities/sudden mood changes (11/28; 39%) (Fig. 3A). Hypotonia (21/28; 75%), scoliosis (18/28; 64%), breastbone abnormalities (15/28; 54%) and advanced bone age during childhood (15/28; 54%) were common musculoskeletal features (Fig. 3B). Another relevant observation included bone fractures, which were reported in a minority of subjects (5/28;18%). Fractures were typically located at the tibia, fibula, and foot. Among these, one individual presented with multiple episodes of tibial fractures (twice on the right tibia and once on the left one) at 2, 4 and 5 years of age. Frequent vision findings were strabismus (16/28; 57%) and vision decline (10/28; 36%) (Fig. 3C). Nine individuals (32%) had optic atrophy; among them, eight presented with a stable condition and one experienced a progression of disease. Refractive disorders (6/28; 21%) included myopia and astigmatism. Among the neurological complications, movement difficulty and problems with coordination were present in more than two thirds of the individuals, and thirteen individuals (46%) had seizures/EEG anomalies (Fig. 3D). Less frequently reported features are presented in Supplementary Table 3 (see Additional file 3).

Additional reported medical problems, involving the cardiovascular and gastrointestinal systems, hearing, sleep and wakefulness, are provided in Supplementary Table 4 (see Additional file 3). More than half of the individuals had constipation (15/28; 54%). Hypersensitivity to noise (13/28; 46%) was frequently reported. Sleep related issues (20/28; 71%) were represented by an increased need for sleep (9/28; 32%), difficulty staying asleep/awaking frequently during night (9/28; 32%), sleep apnea (6/28; 21%) and difficulty falling asleep (4/28; 14%). Among those showing an increased need for sleep, most individuals (7/9; 78%) had an increased need for night-time sleep, while a minority (2/9; 22%) had an increased need for daytime sleep, with additional mood changes occurring when they were not allowed to sleep (4/9; 44%).

Dilated aorta and valve defect were amongst the cardiological complications reported in 8 out 28 subjects (29%). Aortic dilatation was diagnosed at birth and 10 and 30 years of age in the three affected subjects, respectively. A list of medical problems with age of onset or diagnosis reported in adulthood is provided in Table 1.

**Table 1:** Overview of problems with age of onset or diagnosis reported in adulthood in ascending order

Symptom (number of adult individuals)	Age of onset or age at diagnosis (in years)
Optic disc pallor (N = 1)	18
Several psychobehavioral problems such as autistic-like behavior, attention deficit hyperactivity disorder, high levels of frustration and mood abnormalities/disorder (N = 1)	19
Depression (N = 2)	18, 22
Anxiety (N = 3)	18, 19, 20 s
Valve defect (N = 1)	20
Acid reflux (N = 1)	21
Tremors (N = 1)	21
Arthritis (N = 2)	21, 35
Vision decline (N = 3)	20 s, 21, adult-age (exact age not specified)
Obesity (N = 2)	Early adulthood, 23
Decreased mobility (N = 1)	30
Ligament tears (N = 1)	30
Dilated aorta (N = 1)	30
Inflammatory Bowel Disease (N = 1)	35
Sleep problems such as difficulties staying asleep/awaking frequently during the night and increased need for sleep (N = 1)	40 s
Numbness, tingling or painful sensation in muscles (N = 1)	42
Dilated left ventricle (N = 1)	45
Hypertension (N = 1)	45–50
Seizures (N = 3)	18, 23, 49
Cataracts (N = 2)	22, 49

#### Impact on adult quality of life

The medical issues evidenced in the survey had a significant impact on QoL in most of the individuals. The most relevant are reported as follows: psychobehavioral (24/28; 86%), musculoskeletal (22/28; 79%), vision and hearing (22/28; 79%), neurological (18/28; 64%), gastrointestinal (9/28; 32%), sleep (9/28; 32%), respiratory (5/28; 18%) and cardiovascular (4/28; 14%). When considering the severity of symptoms and their influence on QoL, vision problems (12/28; 43%), problems with coordination and movement (12/28; 43%), anxiety (11/28; 39%), and scoliosis (8/28; 29%) were considered as having the most profound impact on adult QoL.

#### Medical, surgical and psychobehavioral treatments

**Fig. 4:**
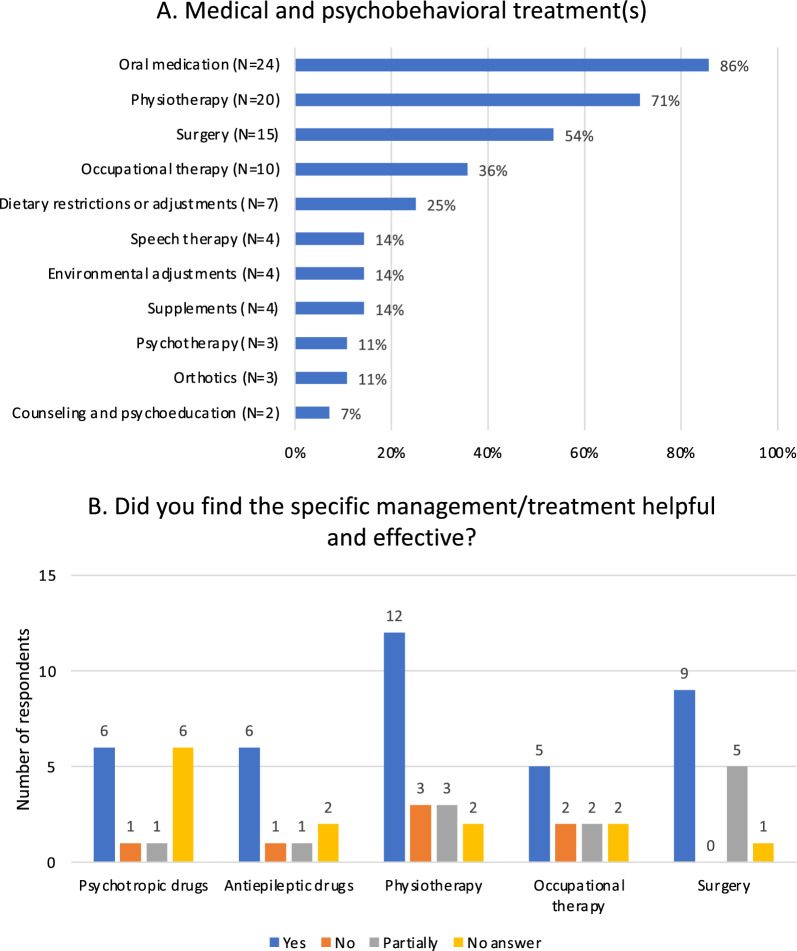
Summary of medical and psychobehavioral treatments. **A**. Type of treatment(s) **B**. Outcome(s) of treatment(s)

A summary of the medical, surgical and psychobehavioral treatments during life and their outcomes is illustrated in Fig. 4A and B, respectively. Medication was taken in 86% of the cases. A list of all medications and their benefits is provided in Supplementary Table 5 (see Additional file 3). The most frequent medications were represented by psychotropic and antiepileptic drugs. Fourteen of the twenty-four individuals (58%) taking medications were taking one or multiple psychotropic drugs. A majority of the subpopulation with EEG anomalies required antiepileptic drug therapy (10/13; 77%). Twenty-three caregivers provided details about required help in taking medication. Two individuals (8.7%) were able to take medication independently, while the majority required assistance. Nine individuals (38%) experienced difficulties with intake and administration, which required crushing of medicines or intake with thickened liquids. Behavioral and intellectual obstacles resulting in inability to properly administer or measure out medication, reluctancy or refusal were reported in 5 individuals (21.7%).

While over 40% of the individuals (12/28) had surgery at least once in adulthood, the problems requiring intervention were generally nonspecific and common in the general adult population. However, spinal surgery seems to be a common intervention among MALNS population. Eighteen individuals presented with scoliosis (64%), nine of which showed a mixed presentation of scoliosis and kyphosis (32%). Nearly half of affected subjects (8/18; 44%) underwent spinal surgery during their life (7 scoliosis and 1 scoliosis/kyphosis), three of these individuals experienced surgical correction during adulthood.

**Fig. 5:**
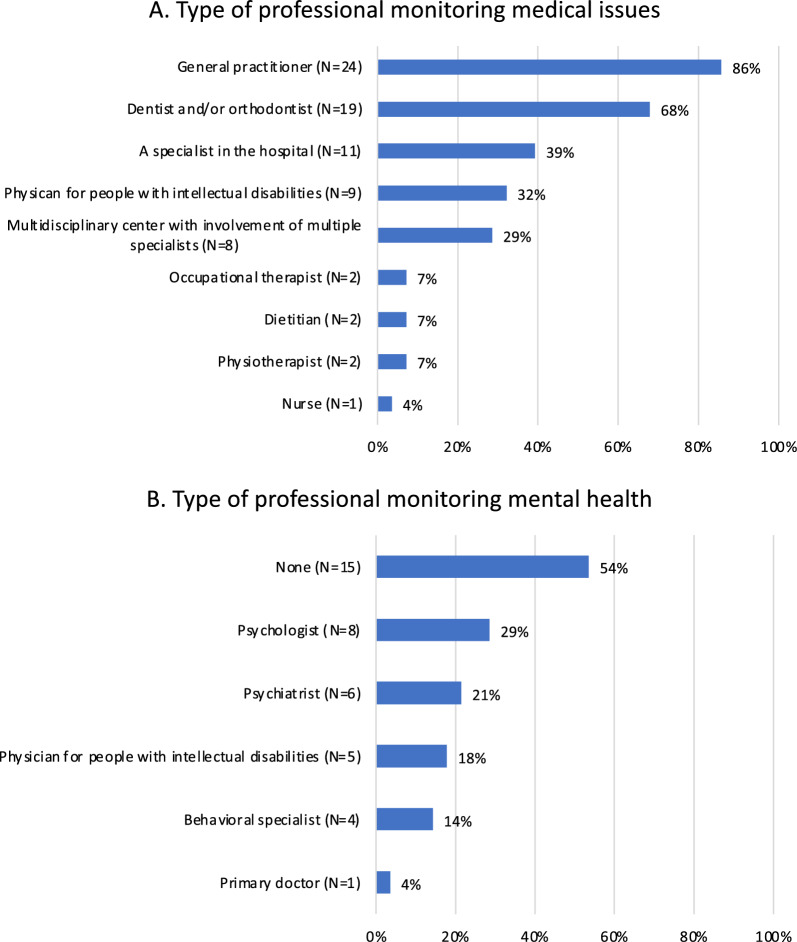
Follow-up in adult individuals with MALNS. **A**. Type of professional monitoring medical issues **B**. Type of professional monitoring mental health

Information was obtained on the types of professional figures who followed up individuals with MALNS either for general medical problems (Fig. 5A) and mental health issues (Fig. 5B). As expected, general practitioners were the most common professional involved in general care and follow up, followed by different specialists on the basis of specific medical problems. Of note, only a minority of individuals (8/28; 29%) were regularly monitored in a multidisciplinary center with expertise in MALNS. This could be related to difficulty in forming a dedicated multi-professional team due to lack of specific expertise on MALNS management, as generally observed for several ultrarare disorders. [19] Frequency of health checks generally ranged from once (18/28; 64%) to twice (4/28; 14%) a year. Periodic follow-up was not considered in the remaining individuals, who required medical consultation in occurrence of specific concerns. While all individuals were monitored for medical issues, strikingly, over half of subjects (15/28; 54%) did not have a regular follow-up for mental health.

### Adult MALNS and SS clinical presentation: similarities and differences

MALNS and SS are two overgrowth conditions that are considered in differential diagnosis. Since its first description, MALNS has been referred to as “Sotos syndrome 2”, although this definition is now outdated and should no longer be used. [6] The currently collected data were compared to the previously reported adult survey in a population of 44 individuals with SS, which was formulated to collect data that were comparable with the present ones, [9], to highlight similarities and differences in the adult populations for the two syndromes and to better aid clinicians with diagnostics and management (Table 2). Several signs significantly differ in terms of prevalence and severity. DD/ID is usually more severe in MALNS with respect to SS (*p* = 0.00194). A significant proportion of individuals with SS has been reported to have normal intellectual development or mild ID (18% to 39%, respectively). Conversely, normal intellectual development has not been reported in MALNS, while mild ID has been rarely observed. [7, 20, 21] Similarly, QoL and daily functioning abilities seem to be more preserved in the SS adult population, especially in those with mild ID who were completely independent in self-caring, better performing and, in most cases, were employed in a protected context. Behavior and psychiatric issues have been reported in a minority of adult individuals with SS (20%). Although the spectrum of presentation (*e.g.*, anxiety, autistiform behavior, anger/aggressive behavior) may be largely overlapping, these features are less represented in the adult SS population compared to the adult MALNS population (*p* = 0.00001). Ophthalmic abnormalities are more common in MALNS in comparison to SS [22]; in particular, optic nerve hypoplasia (ONH) or optic nerve atrophy (ONA) are rare events in SS (*p* = 0.0005). [22, 23] Musculoskeletal anomalies are in general less frequent and severe in SS with the exception of scoliosis. Half of the adults with SS presented with scoliosis and, among them, 46% required surgical correction. A low body mass index (BMI) is rarely observed in SS, as well as a slender habitus with low muscular build (*p* = 0.0024 and *p* = 0.00001, respectively). Of note, use of tube feeding has rarely been reported in MALNS to treat underweight [16] (present data). Cardiac anomalies are observed in SS in 20% of individuals regardless of age, in line with adults with MALNS, which showed a prevalence of 29% (8/28) [24]. Three MALNS individuals out of 28 (11%) presented with aortic dilatation. Of note, four adult individuals with SS have been reported with aortic dilatation as well, (9%) in line with the occurrence of this specific anomaly in MALNS. Aortic dilatation has been also reported in other overgrowth conditions, such as Tatton-Brown-Rahman syndrome [25]. Collectively, these data provide evidence for an association with aortic disease in overgrowth syndromes, and suggest cardiovascular surveillance into adulthood. Finally, four SS female individuals had children. To the best of our knowledge, reproductive fitness in MALNS is extremely low, and there are no reports of MALNS adults with children, although four individuals have been reported to be sexually active (Fig. 2B).

**Table 2:** Clinical features in adult MALNS and SS cohorts

Clinical feature	MALNS—N = 28 (%)	SS—N = 44 (%) [9]	*P* value
Normal intellectual development	Not reported	8/44 (18%)	0.00194
Behavior anomalies	23/28 (82%)	9/44 (20%)	0.00001
Low BMI	6/28 (21%)	Not reported	0.0024
Slender habitus *	46/78 (59%) *	Not reported	0.00001
Visual problems ONH or ONA	26/28 (96%) 9/28 (33%)	2 (sporadic)^#^	0.0005
Cardiac involvement Aortic dilatation	8/28 (30%) 3/28 (11%)	20%^ 4/44 (9%)	n.s n.s
Scoliosis Requiring surgery	18/28 (64%) 8/18 (44%)	24/44 (55%) 11/24 (46%)	n.s n.s
Tremors	6/28 (21%)	3/44 (7%)	n.s

*P* value: Chi-square’s test in 2 × 2 contingency tables (Fisher’s exact test); *from [5] and this study; ^#^two sporadic adult individuals reported in the literature [21, 22]; ^from [23] general SS population; ONH: optic nerve hypoplasia, ONA: Optic nerve atrophy, n.s.: not significant

Based on the present data and previous experience in the pediatric population, individuals with overgrowth and moderate to severe ID, slender habitus with or without low BMI, ophthalmologic issues, musculoskeletal problems (mainly scoliosis) and psychobehavioral issues strongly impacting QoL should be mainly addressed towards a clinical diagnosis of MALNS with respect to SS, although a molecular confirmation through whole exome sequencing or an overgrowth multigene panel including *NFIX, NSD1* and other genes of interest in differential diagnosis is highly recommended.

## Discussion

This is the first study providing data on the natural history and management of MALNS, and its impact on QoL in the largest molecularly confirmed cohort of adult individuals with MALNS (N = 28) from the perspectives of caregivers.

Our data indicate that adult individuals with MALNS may present with different occurrence of some cardinal features of the disorder with respect to childhood and adolescence. MALNS is an overgrowth disorder in which a height higher than two SD is observed in more than half of the children and adolescents [5]. In our cohort, only nine individuals (32%; 5 men/4 women) showed a height higher than two SD. Among these subjects, two had spinal surgery. Our results confirm that overgrowth is less prominent in adulthood. On the other hand, macrocephaly still remains the most distinctive sign of overgrowth in MALNS, as head circumference (OFC) was above two SD in all individuals (N = 16) whose OFC was reported.

This survey also allowed us to highlight some interesting data in adaptive functioning skills with a relevant data in communication abilities in MALNS. As expected by previous reports, [20], all participants indicate impaired language skills in childhood, but the majority of the adults are able to communicate verbally (23/28; 82%). Unexpectedly, 76% of individuals (16/21) showed improvement in speech, meaning that language skills can become better with age. This information suggests partial recovery and/or late improvement of speech, which should be considered when counseling families diagnosed with MALNS in childhood. Adaptive functioning assessment is usually performed with the Vineland adaptive behavior scale—second edition (VABS-II), which specifically explores abilities in three different domains (communication, socialization and daily living skills). Apparent contrasting results for the two other domains (i.e.: socialization and daily living skills) has previously been evidenced between pediatric and adult MALNS populations [20, 26] Specifically, adult MALNS have been found to gradually improve with time in daily living skills eventually reaching an average score later than typical and, in general, performing better than expected [26].

When considering the most frequent medical problems, the clinical presentation in adulthood is mainly characterized by a high prevalence of psychobehavioral comorbidities (96%). They include anxiety (79%), limited interests, repetitive movements and echolalia (50%), autistic traits (46%) and mood abnormalities (39%). These observations show consistency with previous literature, which reported a prevalence between 52 and 94%, mainly represented by anxiety and autistic-like behavior [5, 7, 21]. Notably, despite the high rate, more than half of the individuals (N = 15) were not under direct care and follow-up of mental health professionals. This result may be traced back to several causes. The transition from pediatric to adult healthcare system remains challenging for affected individuals with neurodevelopmental disorders as they (and indirectly their families/caregivers) invariably encounter several obstacles. They are frequently forced to change referring specialists who, in most cases, do not have experience in dealing with rare diseases and their dedicated care. [27, 28] In MALNS individuals, the high anxiety levels in the medical setting and examinations by unfamiliar physicians could lead to less willingness to apply to adult healthcare services. Eventually, these factors put affected individuals at risk of discontinuous and non-adequate support. [27].

In the present cohort, the treatment of psychobehavioral comorbidities was mainly pharmacological (14/27; 52%) whereas only a few individuals received non-pharmacological treatments, such as psychotherapy and psychoeducation (N = 5). A slightly higher pharmacological treatment has been reported in individuals diagnosed with Fragile X syndrome (63%), whereas prevalence of psychotropic usage in Prader-Willi syndrome (37%) appears to be less [29, 30]. Mental health services are more prone to prescribe psychotropic medication, mainly antipsychotics, in adults with ID even in the absence of concurrent psychiatric symptoms [31]. High doses and polypharmacy are also common practice in this population [32]. The use of psychotropic drugs is much higher than in the general population [33]. Based on these clinically relevant findings, we suggest that pharmacological treatment should only be prescribed after proper interdisciplinary assessment, under close monitoring of efficacy and side-effects. Psychological therapy is also suggested to be moderately effective in people with ID. However, concerns regarding lack of experience with this population amongst mental health professionals and the notion that their cognitive problems are a barrier for proper engagement limit the inclusion of psychotherapy in psychobehavioral management [34]. Half of the caregivers reported that drug therapy was effective whereas the other half did not provide an answer (Fig. 4B). This suggests that caregivers find it difficult to define effectiveness of drug therapy and/or how they improved their child’s behavior. The benefit of different psychotropic drugs varies tremendously, which highlights the complexity of behavior in individuals with MALNS as seen in Supplementary Table 5 (see Additional file 3). The importance of a proper psychobehavioral assessment in MALNS has been previously underlined to aid clinicians in timely diagnosis and early intervention at all ages [20, 21]. Our results confirm the usefulness of psychological surveillance also in adulthood. When considering the significant impact of psychobehavioral issues on adult QoL, the need to include a mental health professional in management of adult individuals is highly recommended.

Involvement of the musculoskeletal system is characteristic of MALNS. Previous reports have identified hypotonia (50–76%), abnormal spine curvatures (32–75%), advanced bone age (80%) and flat feet (69%) as common features [5, 7]. We found consistent results regarding hypotonia (75%) and spine anomalies (including scoliosis and/or kyphosis) (64%). These numbers are in line with those in the pediatric cohort [7]. Only half of the adults had an advanced bone age during childhood. As bone age is assessed in children who present with growth abnormalities, those who show overgrowth are more likely to be evaluated and diagnosed [35]. Indeed, there is evidence that advanced bone age is hard to be properly confirmed after early infancy so it might be likely that this specific feature has not been investigated in individuals with MALNS who were molecularly diagnosed in late childhood or adolescence. Five individuals had bone fractures, which mostly occurred during childhood. Despite the small number of associated osteoporosis (N = 3), the increased risk of skeletal fractures suggests that further investigations on bone mineral density in adolescent and adult individuals should be required, as previously reported, [7] and that vitamin D supplementation should be considered.

Problems with coordination was a commonly observed symptom with a higher prevalence compared to the pediatric population. Seventeen individuals (61%) experienced this problem compared to three cases reported in the study by Macchiaiolo et al. (19%). The latter were evaluated by medical professionals and findings were objectified. Moreover, the authors recorded ataxia as sporadic episodes and part of a spectrum of neurovegetative signs [7]. In the present survey, coordination problems were initially defined as “ataxia” and might be misinterpreted by caregivers. The discrepancies between previous data and the present findings can be explained due to this difference in perception by caregivers. They were specifically re- interviewed on this topic, but they could not provide a real distinction between general coordination problems and a medical diagnosis of ataxia. As we cannot objectively verify the occurrence of ataxia in the present adult cohort, it is likely that coordination problems are due, in part, to concomitant visuospatial and visuomotor deficiencies that are invariably observed in MALNS [7, 20].

Seizures/EEG anomalies were present in 46% of the individuals (13/28), while previous studies reported a prevalence of 26.5% and 63% [5, 7]. EEG anomalies were not constantly associated with seizures, in line with previous observations considering other cohorts of individuals with other neurodevelopmental disorders [36, 37]. In the present survey, both features were incorporated into a single question, and therefore could not be separated into two categories. Ten out of thirteen adults (77%) were treated with antiepileptics and were presumed to have developed seizures, whereas the remaining three were asymptomatic. This finding is in contrast with what had previously been reported in the pediatric population [7], which evidenced a high prevalence of EEG anomalies without development of seizures. Overall these findings indicate that late manifestation of seizures should be carefully monitored in adult individuals. In particular, late adult-onset of seizures occurred in three subjects (Table 1), who carried a pathogenic intragenic *NFIX* variant. While, previous reports suggested that deletions involving the *CACNA1A* gene could play a role and potentially increase the risk of developing seizures, [5, 14, 38] the present findings highlight the need for continuous monitoring of possible seizures in adulthood regardless of type of mutation or microdeletion.

Brain abnormalities, such as wide ventricles, corpus callosum hypoplasia, Chiari malformation and brain atrophy have been reported in MALNS [5]. We observed only one individual with Chiari malformation who was diagnosed at 17 years. As we did not ascertain whether all adults have had an MRI nor did we include a separate question in the survey, these features may have been underreported.

The other frequently described features of MALNS in adulthood included visual problems (96%). We observed strabismus in 57% of the adults, comparable with previous reports (63%) [7], all diagnosed in either infancy or childhood. Refractive disorders were less prevalent (21% vs. 75–81%) [5, 7]. We also report relatively high rates of ONA/ONH (32%) and vision decline (10/28; 36%). ONH/ONA are congenital conditions that have been previously reported with a prevalence of 21–25% in pediatric MALNS individuals [5, 7]. Individuals with ONH usually develop nystagmus and strabismus at an early age [39]. ONA was not reported separately before and was grouped under ONH in previous surveys thus we cannot properly verify if there is an increased occurrence in adulthood [5, 7]. ONA refers to irreversible loss of nerve fibers caused by a wide range of diseases and patients often present with a loss of visual field [40]. This might suggest that those with vision decline could potentially have underlying ONA. Over half of the adults (6/10; 60%) with vision decline were also diagnosed with ONA. Three individuals were diagnosed with vision decline in adulthood mainly due to adult-related complications (cataracts and presbyopia), while the rest manifested during childhood. Despite the observed vision decline, no cause was determined in two patients. The exams to determine ONA include visual field tests, magnetic resonance imaging and optical coherence tomography [40]. However due to their anxiety and ID, consultation of ophthalmologists and subsequent examinations may be limited in individuals with MALNS. All these observations, together with the high prevalence of visual impairment in the adult MALNS population, highlights the importance of routine ophthalmologic evaluations at all ages, as previously suggested [7]. This is anticipated to aid in identifying causes that can be accommodated/treated before significant vision loss occurs.

Adults display a lower prevalence of hypersensitivity to noise with respect to children and young adults (46% vs. 67–81%) [7, 21]. This could be explained by the difficulty to secure an accurate diagnosis that is obtained through audiometry to verify the level of discomfort in decibels, together with dedicated questionnaires to assess severity [41]. It is possible that individuals in our cohort have not all been properly evaluated, and the number could be much higher. However, our findings suggest that hypersensitivity to noise might decrease with age.

Other observations include constipation in more than half of the cases (54%). This is consistent with the pediatric prevalence (50%) [7], confirming that it is a significant symptom to adequately treat either early in childhood or in adulthood. Due to ID and language difficulties, it may be challenging for individuals with MALNS to express discomfort or pain. Manifestations of constipation can often present as sleep or behavioral problems [42]. Other gastrointestinal symptoms were not described in previous adult case reports.

We report a high frequency of sleep problems in MALNS (20/28; 71%). This issue seems not to have been reported in the literature. Sleep abnormalities are common in individuals with ID across all ages and impaired sleep is associated with challenging behavior [43]. It is also possible that anxiety may worsen sleep problems, and this could explain the high occurrence of this issue in the adult MALNS population. Based on both high frequencies of constipation and sleep anomalies, we strongly suggest proper evaluation of gastrointestinal and sleep issues in the adults with MALNS who present with psychobehavioral comorbidities.

High pain threshold (46%), skin issues (32%), incontinence (25%), tremors (21%), muscle hypoplasia (18%) and tics (18%) had not previously been reported in MALNS. Three out five adults with tics presented with coexistent seizures for which antiepileptic therapy was required. This might suggest that medication could possibly contribute to the manifestation of tics, as previously reported [44].

A high pain threshold is characteristic of Prader-Willi syndrome caused by dysfunction of the hypothalamus [45]. For MALNS it remains unclear whether the parents’ reported high pain threshold is consistent with a higher threshold point at which a stimulus becomes painful or that it underlies altered pain tolerance or reactions. As mentioned before, individuals with ID do not always provide reliable self-reporting of pain and have to rely on their caregivers to observe behavior that indicates pain [46]. Children and adolescents with ID display a higher prevalence of incontinence [47]. It is possible that there is a correlation between pain perception and incontinence as affected individuals might be less aware of the sensory signals of a full bladder. Caregivers and professionals should keep an altered pain perception in mind when evaluating any medical issues.

Skin issues could not be properly specified to obtain possible occurrence of dermatologic conditions. They generally occurred during childhood. Therefore, an age-related association seems less likely. A recurrent finding were eczemas, which are a relatively common in the general population [48].

Six individuals presented with “tremors” as an additional feature with variable onset varied (from birth to adulthood) and possibly related to different triggering events, including anti-psychotic drugs. Follow-up with a neurologist is desired to accurately objectify these tremors and further examine associations with psychobehavioral comorbidities and seizures.

A diminished volume in muscle tissue has been observed in a minority of individuals (5/28; 18%). This sign is invariably associated with low BMI (see below). Although we do not have a clear explanation about this feature, we might hypothesize a connection between the two signs. *NFIX* plays an important role in skeletal muscle development, regulating the switch from embryonic to fetal myogenesis by specifically activating fetal genes [49]. This crucial role has been also established in the mouse model of the disorder in which an inhibitory mechanism at the promoter of the gene that encodes for myostatin*,* a TGF-β family member with anti-myogenic properties, has been evidenced [50]. This finding is consistent with the hypothesis that MALNS individuals could show a reduced muscular mass due to the inability to gain weight despite adequate nutrient intake [7]. On the other hand, it might be possible that a diminished muscular mass might be also related to decreased mobility and diminished physical activity due to movement and coordination problems, which are frequently observed in these subjects. Further studies are needed to confirm a possible concomitant effect of these causes on muscle volume in MALNS.

Our survey evidenced some age-related signs diagnosed at a younger age than normally expected. We observed three individuals affected with cataracts at 4, 13 and 22 years of age. Cataract surveillance should be performed at all ages in MALNS population due to possible anticipation of early-onset in these individuals.

The oldest individual (60 years old) presented with a relatively stable health and reported adult-onset features, such as presbyopia, dilated left ventricle and hypertension. Signs such as acid reflux and inflammatory bowel disease were also sporadically reported in adulthood. Again, these issues might not be directly related to MALNS, as these are complications statistically normally occurring in the general adult population [51–53]. Cancer, macular degeneration, and stroke were not reported.

We observed obesity in three individuals (11%), which is in contrast to slender habitus and low BMI typical of pediatric MALNS [5, 7]. Underweight/low BMI was also observed in six adult individuals (21%). Among them, one individual required nasogastric tube feeding and involvement of a dietitian. Tube feeding-dependence had previously been reported [16]. These observations indicate that weight and BMI should be evaluated at every follow-up appointment, and stress the importance of sufficient calorie intake, with the potential help of a dietitian, to prevent complications and possibly avoid tube feeding.

MALNS is currently not classified as a disorder predisposing for cardiovascular disease. Previous series have reported dilatation of large blood vessels; among them, one had dilatation of the pulmonary arteries and five presented with dilatation of the aorta [5, 13, 54]. One individual showed progression of the aortic dilatation between 35 and 38 years of age with dissection [13]. Macchiaiolo et al. [7] evaluated 16 pediatric individuals with echocardiography and only observed mitral regurgitation in 31%. We report three individuals with aortic dilatation diagnosed at various ages (birth, 10 years, and 30 years). Clinical significance and progression of dilatation cannot be determined yet, but these observations signify the importance of cardiological evaluation in all affected individuals at diagnosis and follow-up.

### Preliminary dataset of recommendations for management and follow-up in adulthood

The 1-year surveillance study by Macchiaiolo et al. provided a set of recommendations for management and follow-up of all individuals with MALNS [7]. Based on the presently collected data, we propose additional recommendations focused on adulthood (Table 3).

**Table 3:** Recommendations for management and follow-up in MALNS adulthood

Type of evaluation	Evaluation, frequency, and follow-up from Macchiaiolo et al. [7]	Evaluation, frequency, and follow-up in adulthood
Auxological evaluation	Annual evaluation of weight, height, head circumference. Calculate BMI-SDS and calories intake	Continue annual evaluation in adulthood Pay attention to weight gain in case of psychotropic drug therapy Refer to nutritionist in case of (risk of) underweight
Orthopedic evaluation	Evaluate spine curvature, body length discrepancies, flat feet. at diagnosis, then evaluate annually until puberty Accurate anamnesis for bone fractures. Consider DXA	Continue annual evaluation in adulthood Consider evaluation of bone mineral density with DXA-scan
Ophthalmologic evaluation	Search for refractive errors, nystagmus, strabismus, cataract, and optic disk pallor at diagnosis, and then annually until puberty	Continue annual evaluation and eye examination in adulthood Be aware of optic nerve atrophy and early-onset cataracts Refer to appropriate services as early as possible for assistive devices
Psychobehavioral evaluation	Perform neuropsychiatric and behavior evaluation at diagnosis, then on neuropsychiatric indication	Annual behavior evaluation. On neuropsychiatric indication, consider psychotropic drugs and side effects Continue follow-up in all patients by specialized practitioners (psychologist, psychiatrist, behavioral specialist) Consider secondary causes of changed behavior: constipation and sleep problems
Cardiological evaluation	At diagnosis, refer to pediatrician for periodic clinical evaluation	General practitioner monitors blood pressure and performs physical examination annually Annual or biennial evaluation by cardiologist with repeat echocardiogram as needed for aortic dilatation
Neurological evaluation	If EEG aspecific anomalies alone are detected: watch and wait strategy. Patients with microdeletions consider closer follow-up	Periodic evaluation by neurologist as needed Consider more frequent follow-up in adulthood due to possible late manifestations of seizures regardless of type of mutation or microdeletions
Gastrointestinal evaluation	Look for constipation and treat it	Annual or twice a year evaluation
Urological evaluation	–	Evaluate toileting habits, paying attention to incontinence
Sleep evaluation	–	Pay attention to sleep health and sleep disorders (sleep apnea)
Cancer surveillance	No evidence for strict surveillance	Cancer surveillance as recommended by local adult guidelines (breast, cervical and colon)

### Strengths and limitations

Strengths of this study include close collaboration with the international patient advocacy group, the Malan Syndrome Foundation, and the Italian and Dutch MALNS centers of expertise, which allowed easy access to recruit all known caregivers of adult individuals with MALNS. Additionally, combined input from these organized entities has helped to interpret open answers and establish uniform definition of symptoms.

In general, we observed a high response rate of 18/28 adult individuals (64%) who were registered at the Malan Syndrome Foundation. Despite the small number of participants, the present cohort is representative with respect to the prevalence of this ultra-rare disorder. The adapted survey was extensive, and questions covered a wide range of aspects of health, behavior, daily functioning, and treatments. The cross-sectional approach allowed us to quickly collect data in a short period of time and establish prevalence of multiple signs and symptoms. However, this approach also has some limitations such as the inability to objectively assess signs and symptoms and determine their prevalence. There is also recall bias risk as participants may not always be able to remember information correctly. We also had some missing data, mostly concerning age of onset or diagnosis and, type and outcomes of treatments, as not all questions were required to be answered.

## Conclusions

Collecting information in the adult population has allowed a more complete description of the natural history of MALNS. Individuals with MALNS are burdened with psychobehavioral comorbidities, musculoskeletal problems, vision impairment and neurological issues that persist in adulthood, have a significant impact on QoL and require life-long support and health monitoring. A possible partial recovery of communication abilities in adults should be considered in genetic counseling of families diagnosed with MALNS in childhood. The comparison of MALNS and SS adult presentation highlights similarities and differences to clinically guide physicians with proper diagnostics and management. Future research should focus on further delineation of the genotype–phenotype correlation in a longitudinal setting to accurately assess incidence of symptoms and progression of the disorder. Findings will contribute to the development of standards for clinical evaluation and physical and mental health management in adults with MALNS to assure optimal monitoring and treatment of possible evolutive complications.

### Supplementary Information

**Additional file 1**. English Malan syndrome adult survey.

**Additional file 2**. Supplemental material: table 1 literature review of adults with Malan syndrome.

**Additional file 3**. Supplemental material: Tables 2-5 and Figures 1-2.

## Data Availability

All data generated or analyzed during this study are included in this published article and its supplementary files.

## References

[1] Claussnitzer M, Cho JH, Collins R, Cox NJ, Dermitzakis ET, Hurles ME, et al. A brief history of human disease genetics. Nature. 2020;577(7789):179–189. 10.1038/s41586-019-1879-7 PMC7405896 31915397

[2] Parenti I, Rabaneda LG, Schoen H, Novarino G. Neurodevelopmental disorders: from genetics to functional pathways. Trends Neurosci 2020;43(8):608–621. 10.1016/j.tins.2020.05.004 32507511

[3] Dykens EM. Aging in rare intellectual disability syndromes. Dev Disabil Res Rev. 2013;18(1):75–83. 23949831 10.1002/ddrr.1130

[4] Orphanet: Malan overgrowth syndrome. Available at: https://www.orpha.net/consor/cgi-bin/OC_Exp.php?lng=EN&Expert=420179. Accessed Mar 29, 2023.

[5] Priolo M, Schanze D, Tatton-Brown K, Mulder PA, Tenorio J, Kooblall K, et al. Further delineation of Malan syndrome. Hum Mutat. 2018;39(9):1226–37. 29897170 10.1002/humu.23563 PMC6175110

[6] Malan V, Rajan D, Thomas S, Shaw AC, Louis Dit Picard H, Layet V, et al. Distinct effects of allelic NFIX mutations on nonsense-mediated mRNA decay engender either a Sotos-like or a Marshall-Smith syndrome. Am J Hum Genet 2010;87(2):189–198. 10.1016/j.ajhg.2010.07.001 PMC2917711 20673863

[7] Macchiaiolo M, Panfili FM, Vecchio D, Gonfiantini MV, Cortellessa F, Caciolo C, et al. A deep phenotyping experience: up to date in management and diagnosis of Malan syndrome in a single center surveillance report. Orphanet J Rare Dis. 2022;17(1):235. 35717370 10.1186/s13023-022-02384-9 PMC9206304

[8] Baas M, Huisman S, van Heukelingen J, Koekkoek G, Laan H, Hennekam RC. Building treasures for rare disorders. Eur J Med Genet 2015;58(1):11–13. 10.1016/j.ejmg.2014.10.006 25449139

[9] Foster A, Zachariou A, Loveday C, Ashraf T, Blair E, Clayton-Smith J, et al. The phenotype of Sotos syndrome in adulthood: a review of 44 individuals. Am J Med Genet. 2019;181(4):502–8. 31479583 10.1002/ajmg.c.31738

[10] Douzgou S, Dell’Oro J, Fonseca CR, Rei A, Mullins J, Jusiewicz I, et al. The natural history of adults with Rubinstein-Taybi syndrome: a families-reported experience. Eur J Hum Genet 2022;30(7):841–847. 10.1038/s41431-022-01097-8 PMC9259744 35388185

[11] Rare Disease Registry. Sanford Research. Available at: https://research.sanfordhealth.org/rare-disease-registry. Accessed May 20, 2023.

[12] Yoneda Y, Saitsu H, Touyama M, Makita Y, Miyamoto A, Hamada K, et al. Missense mutations in the DNA-binding/dimerization domain of NFIX cause Sotos-like features. J Hum Genet 2012;57(3):207–211. 10.1038/jhg.2012.7 22301465

[13] Oshima T, Hara H, Takeda N, Hasumi E, Kuroda Y, Taniguchi G, et al. A novel mutation of NFIX causes Sotos-like syndrome (Malan syndrome) complicated with thoracic aortic aneurysm and dissection. Human Genome Var. 2017;4:17022. 10.1038/hgv.2017.22 PMC5451486 28584646

[14] Bellucco FT, de Mello CB, Meloni VA, Melaragno MI. Malan syndrome in a patient with 19p13.2p13.12 deletion encompassing NFIX and CACNA1A genes: case report and review of the literature. Mol Genet Genom Med 2019;7(12):e997. 10.1002/mgg3.997 PMC6900369 31574590

[15] Hancarova M, Havlovicova M, Putzova M, Vseticka J, Prchalova D, Stranecky V, et al. Parental gonadal but not somatic mosaicism leading to de novo NFIX variants shared by two brothers with Malan syndrome. Am J Med Geneti A 2019;179(10):2119–2123. 10.1002/ajmg.a.61302 31369202

[16] Sihombing NRB, Winarni TI, van Bokhoven H, van der Burgt I, de Leeuw N, Faradz SMH. Pathogenic variant in NFIX gene affecting three sisters due to paternal mosaicism. Am J Med Genet A 2020;182(11):2731–2736. 10.1002/ajmg.a.61835 32945093

[17] Priolo M, Grosso E, Mammì C, Labate C, Naretto VG, Vacalebre C, Caridi P, Laganà C. A peculiar mutation in the DNA-binding/dimerization domain of NFIX causes Sotos-like overgrowth syndrome: a new case. Gene. 2012;511(1):103–5. 22982744 10.1016/j.gene.2012.08.040

[18] Gurrieri F, Cavaliere ML, Wischmeijer A, Mammì C, Neri G, Pisanti MA, et al. NFIX mutations affecting the DNA-binding domain cause a peculiar overgrowth syndrome (Malan syndrome): a new patients series. Eur J Med Genet. 2015;58(9):488–91. 26193383 10.1016/j.ejmg.2015.06.009

[19] Van Groenendael S, Giacovazzi L, Davison F, Holtkemper O, Huang Z, Wang Q, et al. High quality, patient centred and coordinated care for Alstrom syndrome: a model of care for an ultra-rare disease. Orphanet J Rare Dis 2015;10:149. 10.1186/s13023-015-0366-y PMC4657378 26603037

[20] Alfieri P, Macchiaiolo M, Collotta M, Montanaro FAM, Caciolo C, Cumbo F, et al. Characterization of cognitive, language and adaptive profiles of children and adolescents with Malan syndrome. J Clin Med 2022;11(14). 10.3390/jcm11144078 PMC9316998 35887841

[21] Alfieri P, Montanaro FAM, Macchiaiolo M, Collotta M, Caciolo C, Galassi P, et al. Behavioral profiling in children and adolescents with Malan syndrome. Front Child Adolescent Psychiatry 2023. 10.3389/frcha.2023.1106228 PMC11732151 39816865

[22] Inoue K, Kato S, Numaga J, Sakurai M, Ohara C, Ouchi M, et al. Optic disk pallor and retinal atrophy in Sotos syndrome (cerebral gigantism). Am J Ophthalmol 2000;130(6):853–854. 10.1016/s0002-9394(00)00711-x 11124319

[23] Nalini A, Biswas A. Sotos syndrome: an interesting disorder with gigantism. Ann Indian Acad Neurol 2008;11(3):190–192. 10.4103/0972-2327.42941 PMC2771975 19893668

[24] Tatton-Brown K, Cole TR, Rahman N. Sotos Syndrome. In: Adam MP, Mirzaa GM, Pagon RA, Wallace SE, Bean LJ, Gripp KW, et al, editors. GeneReviews® Seattle (WA): University of Washington, Seattle; 1993. 20301652

[25] Cecchi AC, Haidar A, Marin I, Kwartler CS, Prakash SK, Milewicz DM. Aortic root dilatation and dilated cardiomyopathy in an adult with Tatton-Brown-Rahman syndrome. Am J Med Genet A 2022;188(2):628–634. 10.1002/ajmg.a.62541 PMC9175539 34644003

[26] Mulder PA, van Balkom IDC, Landlust AM, Priolo M, Menke LA, Acero IH, et al. Development, behaviour and sensory processing in Marshall-Smith syndrome and Malan syndrome: phenotype comparison in two related syndromes. J Intell Disabil Res JIDR. 2020;64(12):956–69. 10.1111/jir.12787 PMC8957705 33034087

[27] Antolini G, Colizzi M. Where do neurodevelopmental disorders go? Casting the eye away from childhood towards adulthood. Healthcare 2023;11(7):1015. 10.3390/healthcare11071015 PMC10094062 37046942

[28] Brown M, Macarthur J, Higgins A, Chouliara Z. Transitions from child to adult health care for young people with intellectual disabilities: a systematic review. J Adv Nurs. 2019;75(11):2418–34. 30816570 10.1111/jan.13985

[29] Dominick KC, Andrews HF, Kaufmann WE, Berry-Kravis E, Erickson CA. Psychotropic drug treatment patterns in persons with fragile X syndrome. J Child Adolesc Psychopharmacol. 2021;31(10):659–69. 34818076 10.1089/cap.2021.0042

[30] Sinnema M, Maaskant MA, van Schrojenstein Lantman-de Valk, Henny M. J., Boer H, Curfs LMG, Schrander-Stumpel CTRM. The use of medical care and the prevalence of serious illness in an adult Prader–Willi syndrome cohort. European J Med Genet 2013;56(8):397–403. 10.1016/j.ejmg.2013.05.011 23792791

[31] Koch AD, Dobrindt J, Schützwohl M. Prevalence of psychotropic medication and factors associated with antipsychotic treatment in adults with intellectual disabilities: a cross‐sectional, epidemiological study in Germany. J Intellectual Disabil Res 2021;65(2):186–198. 10.1111/jir.12802 33393123

[32] Bowring DL, Totsika V, Hastings RP, Toogood S, McMahon M. Prevalence of psychotropic medication use and association with challenging behaviour in adults with an intellectual disability. A total population study. Journal of Intellectual Disability Research 2017;61(6):604–617. 10.1111/jir.12359 28090687

[33] Ohayon MM, Lader MH. Use of psychotropic medication in the general population of France, Germany, Italy, and the United Kingdom. J Clin Psychiatry. 2002;63(9):817–25. 12363124 10.4088/JCP.v63n0912

[34] Vereenooghe L, Langdon PE. Psychological therapies for people with intellectual disabilities: a systematic review and meta-analysis. Res Dev Disabil. 2013;34(11):4085–102. 24051363 10.1016/j.ridd.2013.08.030

[35] Spadoni GL, Cianfarani S. Bone age assessment in the workup of children with endocrine disorders. Horm Res Paediatr. 2010;73(1):2–5. 20190534 10.1159/000271910

[36] Wang J, Ethridge LE, Mosconi MW, White SP, Binder DK, Pedapati EV, et al. A resting EEG study of neocortical hyperexcitability and altered functional connectivity in fragile X syndrome. J Neurodev Disord. 2017;9(1):11. 28316753 10.1186/s11689-017-9191-z PMC5351111

[37] Nicotera AG, Hagerman RJ, Catania MV, Buono S, Di Nuovo S, Liprino EM, et al. EEG abnormalities as a neurophysiological biomarker of severity in autism spectrum disorder: a pilot cohort study. J Autism Dev Disord. 2019;49(6):2337–47. 30726535 10.1007/s10803-019-03908-2

[38] Li X, Li Z, Liang X, Liu D, Jiang M, Gao L, et al. CACNA1A mutations associated with epilepsies and their molecular sub-regional implications. Front Mol Neurosci. 2022;15: 860662. 35600082 10.3389/fnmol.2022.860662 PMC9116572

[39] Garcia-Filion P, Borchert M. Optic nerve hypoplasia syndrome: a review of the epidemiology and clinical associations. Curr Treat Options Neurol. 2013;15(1):78–89. 23233151 10.1007/s11940-012-0209-2 PMC3576022

[40] Ahmad SS, Kanukollu VM. Optic Atrophy. StatPearls Treasure Island: StatPearls Publishing; 2023. 32644556

[41] Aazh H, Knipper M, Danesh AA, Cavanna AE, Andersson L, Paulin J, et al. Insights from the third international conference on hyperacusis: causes, evaluation, diagnosis, and treatment. Noise Health 2018;20(95):162–170. 10.4103/nah.NAH_2_18 PMC6122267 30136676

[42] Robertson J, Baines S, Emerson E, Hatton C. Prevalence of constipation in people with intellectual disability: a systematic review. J Intellect Dev Disabil. 2018;43(4):392–406. 10.3109/13668250.2017.1310829 29194030

[43] van de Wouw E, Evenhuis HM, Echteld MA. Prevalence, associated factors and treatment of sleep problems in adults with intellectual disability: a systematic review. Res Dev Disabil. 2012;33(4):1310–32. 22502859 10.1016/j.ridd.2012.03.003

[44] Peters J, Vijiaratnam N, Angus-Leppan H. Tics induced by antiepileptic drugs: a pragmatic review. J Neurol. 2021;268(1):321–36. 32804278 10.1007/s00415-020-10153-6

[45] Butler MG, Victor AK, Reiter LT. Autonomic nervous system dysfunction in Prader–Willi syndrome. Clin Autonom Res. 2022. 10.1007/s10286-022-00909-7 36515769

[46] Findlay L, Williams ACdC, Baum S, Scior K. Caregiver Experiences of Supporting Adults with Intellectual Disabilities in Pain. J Appl Res Intellect Disabil 2015;28(2):111–120. 10.1111/jar.12109 24909927

[47] von Gontard A, Hussong J, Yang SS, Chase J, Franco I, Wright A. Neurodevelopmental disorders and incontinence in children and adolescents: Attention-deficit/hyperactivity disorder, autism spectrum disorder, and intellectual disability—A consensus document of the International Children’s Continence Society. Neurourol Urodyn. 2022;41(1):102–14. 34586694 10.1002/nau.24798

[48] Langan SM, Mulick AR, Rutter CE, Silverwood R, Asher I, García-Marcos L, et al. Trends in eczema prevalence in children and adolescents: A Global Asthma Network Phase I Study. Clinical and Experimental Allergy 2023.

[49] Messina G, Biressi S, Monteverde S, Magli A, Cassano M, Perani L, et al. Nfix regulates fetal-specific transcription in developing skeletal muscle. Cell 2010;140(4):554–566. 10.1016/j.cell.2010.01.027 20178747

[50] Rossi G, Antonini S, Bonfanti C, Monteverde S, Vezzali C, Tajbakhsh S, et al. Nfix regulates temporal progression of muscle regeneration through modulation of myostatin expression. Cell Rep 2016;14(9):2238–2249. 10.1016/j.celrep.2016.02.014 PMC4793149 26923583

[51] Mills KT, Stefanescu A, He J. The global epidemiology of hypertension. Nat Rev Nephrol 2020;16(4):223–237. 10.1038/s41581-019-0244-2 PMC7998524 32024986

[52] Yamasaki T, Hemond C, Eisa M, Ganocy S, Fass R. The changing epidemiology of gastroesophageal reflux disease: are patients getting younger. J Neurogastroenterol Motil. 2018; 24(4):559–569. 10.5056/jnm18140 PMC6175565 30347935

[53] Ye Y, Manne S, Treem WR, Bennett D. Prevalence of inflammatory bowel disease in pediatric and adult populations: recent estimates from large national databases in the United States, 2007–2016. Inflamm Bowel Dis. 2019;26(4):619–25. 10.1093/ibd/izz182 31504515

[54] Nimmakayalu M, Horton VK, Darbro B, Patil SR, Alsayouf H, Keppler-Noreuil K, et al. Apparent germline mosaicism for a novel 19p13.13 deletion disrupting NFIX and CACNA1A. Am J Med Genet 2013;161(5):1105–1109. 10.1002/ajmg.a.35790 23495138

