# Supplementary material for: Natural history in Malan syndrome: survey of 28 adults and literature review
Source: Orphanet J Rare Dis. 2024 Jul 29;19:282. doi: 10.1186/s13023-024-03288-6 (PMC11288048; doi:10.1186/s13023-024-03288-6)
Supplement: Supplementary file 1 — Additional file 1. English Malan syndrome adult survey. [file 13023_2024_3288_MOESM1_ESM.docx]

**Additional file 1 - Natural history in Malan syndrome: survey of 28 adults and literature review**

**INTRODUCTION**

In collaboration with the Malan Syndrome Foundation, Amsterdam University Medical Centers (Amsterdam UMC) is conducting a research study in which we collect information about health, behaviour, daily functioning, needs and clinical management of adult individuals (18 years and older) with Malan syndrome (MALNS).

This information is crucial for understanding the natural history of MALNS: the full spectrum of symptoms and how the disorder affects individuals over time. Furthermore, this information helps to answer questions of individuals with MALNS and their families, and to develop management guidelines for optimal care.

You are being invited to participate in this research study. We would be grateful if you would fill in the following survey.

- Participation is voluntary. By completing the survey, you are voluntarily agreeing to participate.
- None of these questions are required but your complete responses will be of great help.
- As it is most likely that a caregiver may answer the questions, we worded these accordingly.
- The survey takes approximately 30-45 minutes to complete. You can pause the survey and answers will be saved. You can resume the survey at any given moment.
- If you have any remaining questions about the study, please contact: [t.n.huynh@amsterdamumc.nl](mailto:t.n.huynh@amsterdamumc.nl) OR [christal@malansyndrome.org](mailto:christal@malansyndrome.org)

Please read the instructions below carefully

- There should only be ONE respondent for each adult with MALNS.
- We kindly ask you to complete this survey before 8*^th^ May 2023.*
- Please review your answers before submitting them, to ensure they are complete and correct to the greatest extent possible.
- At the end of the survey, do not exit but click on ‘Finish Survey’ to submit your answers.

**PERMISSIONS & DATA SHARING**

- I voluntarily agree to participate in this study.
- I give permission to Amsterdam UMC to provide and use my information that may or may not be identifiable for research including publication and education purposes.
- I give permission to Amsterdam UMC to share my information with the Malan Syndrome Foundation for non-research purposes.
- I have already filled in the survey of the Sanford CoRDS Patient Registry and I give permission to use my information.
  If YES, please state your participant ID (if unknown, please leave it blank)
  _____________________ *(free text answer)*
- I give permission on …. DATE OF TODAY….

1. GENERAL QUESTIONS
2. Are you a caregiver or the person with Malan syndrome? (Selec*t one*)
   1. Caregiver
   2. Person with Malan syndrome
3. If you answered ‘Caregiver’ in Question 1, could you specify (for example mother, father, sibling, professional caregiver, etc)

________­­­­­­____________­­ (*free text answer*)

1. How old is the person with Malan syndrome?

____________________ (*free text answer*)

4. What is the gender of the person with Malan syndrome? (Select *one*)

a. Female

b. Male

c. Other

d. Prefer not to answer

5. Country of residence:

___________________ (*free text answer*)

1. Where does the person with Malan syndrome currently live?
   1. At home with primary caregiver(s) full-time (for example family)
   2. Assisted living homes: a living arrangement with support from professional caregivers for everyday tasks.
   3. On their own/independently
   4. Other
2. If you answered ‘Other’ in Question 6, could you specify?

_____________________ (*free text answer*)

1. Current height: __________ *(free text answer)*
2. Current weight: __________ *(free text answer)*
3. Current head circumference: __________ *(free text answer)*
4. At what age was the person diagnosed with Malan syndrome? (Select *one*)
   1. Newborn/Infancy (less than 1 year old)
   2. 1-5 years old
   3. 6-10 years old
   4. Older than 10 years
   5. I do not know
5. Has the Malan syndrome diagnosis been confirmed with genetic testing? (Select *one*)
   1. Yes
      If YES, please upload the results of genetic testing/diagnosis. You can upload a photo or file (Word, PDF). Please make sure to leave out any personal information.
   2. No
   3. I do not know
6. Does the person with Malan syndrome have any of the following medical problems that have impacted the quality of life in ADULTHOOD? (*Select all that apply*)
   1. Yes, neurological problems
   2. Yes, musculoskeletal problems
   3. Yes, cardiovascular problems
   4. Yes, respiratory problems
   5. Yes, gastrointestinal problems
   6. Yes, sleep problems
   7. Yes, vision and/or hearing problems
   8. Yes, behavioural/psychiatric problems
   9. Yes, other problems (not mentioned above)
   10. No, none of the above

1. NEUROLOGICAL PROBLEMS
2. If the person with Malan syndrome currently has or has had neurological problems in ADULTHOOD, can you find them in the options below? (*select all that apply*)
   1. Problems with coordination (ataxia)
   2. Migraine with temporary weakness on one side of the body (hemiplegic migraine)
   3. Seizures/EEG anomalies
   4. Stroke
   5. Sudden twitches or fast, repetitive muscle movements (tics)
   6. Chiari malformation
   7. Other
   8. Unsure
   9. No problems
3. If you answered ‘Other’ in Question 14, please specify.

_______________________________________ (*free text answer*)

1. If you chose one or more problems in Question 14, please indicate the age when each neurological problem started/was diagnosed.
   _______________________________________ (*free text answer*)
2. If you chose one or more options in Question 14, what specific management/treatment was used? Please specify per problem.

_______________________________________ (*free text answer*)

1. If you filled in the previous question, did you find the specific management/treatment helpful and effective? Please specify per treatment.

_______________________________________ (*free text answer*)

1. If you answered ‘Seizures/EEG anomalies’ in Question 14 and ‘antiepileptic drugs (AED)/medication’ in Question 17, please specify which AED the person with Malan syndrome CURRENTLY takes? (*Select all that apply*)
   1. Brivaracetam
   2. Carbamazepine
   3. Gabapentin
   4. Keppra (Levetiracetam)
   5. Lamotrigine
   6. Topiramate
   7. Trileptal (Oxacarbazepine)
   8. Other
      Please specify _____________________________ *(free text answer)*
2. If you selected any options in Question 14, which of the neurological problems have the MOST impact on the quality of life in ADULTHOOD? Please specify.

______________________________________________ *(free text answer)*

1. MUSCULOSKELETAL PROBLEMS
2. If the person with Malan syndrome currently has or has had muscular problems in ADULTHOOD, can you find them in the options below? (*Select all that apply*)
   1. Coordination and movement difficulty
   2. Droopy eyelids
   3. Excessive drooling
   4. Low muscle tone (hypotonia)
   5. Muscle loss (atrophy)
   6. Numbness, tingling or painful sensation in muscles.
   7. Twisting of neck to one side (torticollis)
   8. Trouble with breathing
   9. Trouble with swallowing
   10. Other
   11. Unsure
   12. No problems
3. If you answered ‘Other’ in Question 21, please specify.

_____________________________________ (*free text answer*)

1. If you chose one or more options in Question 21, please indicate the age when each muscular problem started/was diagnosed.
   _____________________________________ (*free text answer*)
2. If you chose one or more options in Question 21, what specific management/treatment was used? Please specify per problem.

______________________________________ (*free text answer*)

1. If you filled in the previous question, did you find the specific management/treatment helpful and effective? Please specify per treatment.

______________________________________ *(free text answer)*

1. Which of the following skeletal features does the person with Malan syndrome exhibit? (*Select all that apply*)
   1. Advanced bone age
   2. Breastbone cave inward (pectus excavatum)
   3. Breastbone cave outward (pectus carinatum)
   4. Bone fractures
   5. Curved legs (tibial bowing)
   6. Excessive outward curvature of the spine (kyphosis)
   7. Excessive inward curvature of the spine (lordosis)
   8. Hip deformity (coxa valga)
   9. Osteoporosis
   10. Pelvic bone abnormality
   11. Rib malformations
   12. Sideways curvature of the spine (scoliosis)
   13. Other
   14. Unsure
   15. No problems
2. If you answered ‘Other’ in Question 26, please specify.

________________________________________ (*free text answer*)

1. If you chose one or more options in Question 26, please indicate the age when each skeletal problem started/was diagnosed.

_________________________________________ *(free text answer)*

1. If you chose one or more options in Question 26, what specific management/treatment was used? Please specify per problem.

__________________________________________ (*free text answer*)

1. If you filled in the previous question, did you find the specific management/treatment helpful and effective? Please specify per treatment.
   __________________________________________ *(free text answer)*
2. If you answered ‘Bone fractures’ in Question 26, how many fractures has the person with Malan syndrome had to date and in which bone/location? Please specify per fracture.

__________________________________________ (*free text answer)*

1. If you selected any options in Question 21 and/or Question 26, which of the musculoskeletal problems have the MOST impact on the quality of life in ADULTHOOD? Please specify.

__________________________________________ *(free text answer)*

1. CARDIOVASCULAR PROBLEMS
2. If the person with Malan syndrome currently has or has had cardiovascular problems in ADULTHOOD, can you find them in the options below? (*select all that apply*)
   1. Dilated aorta
   2. Dilated pulmonary artery
   3. Heart murmur
   4. High blood pressure
   5. Low blood pressure
   6. Valve defect
   7. Other
   8. Unsure
   9. No problems
3. If you answered ‘Other’ in Question 33, please specify.

______________________________________ (*free text answer*)

1. If you chose one or more options in Question 33, please indicate the age when each cardiovascular problem was diagnosed.
   ______________________________________ (*free text answer*)
2. If you chose one or more options in Question 33, what specific management/treatment was used? Please specify per problem.

_______________________________________ (*free text answer*)

1. If you filled in the previous question, did you find the specific management/treatment helpful and effective? Please specify per treatment.

_______________________________________ *(free text answer)*

1. If you selected any options in Question 33, which of the cardiovascular problems have the MOST impact on the quality of life in ADULTHOOD? Please specify.

____________________________________________ *(free text answer)*

1. RESPIRATORY PROBLEMS
2. If the person with Malan syndrome currently has or has had respiratory problems in ADULTHOOD, can you find them in the options below? (*select all that apply*)
   1. Acute bronchitis
   2. Asthma
   3. Abnormal enlargement of airways of the lungs (bronchiectasis)
   4. Full/partial lung collapse
   5. Pulmonary edema
   6. Pulmonary embolus
   7. Pulmonary fibrosis
   8. Recurrent pneumonia
   9. Reduced lung capacity
   10. Other
   11. Unsure
   12. No problems
3. If you answered “Other” in Question 39, please specify.

______________________________________(*free text answer*)

1. If you chose one or more options in Question 39, please indicate the age when each respiratory problem started/was diagnosed.
   _____________________________________ (*free text answer*)
2. If you chose one or more options in Question 39, what specific management/treatment was used? Please specify per problem.

______________________________________ (*free text answer*)

1. If you filled in the previous question, did you find the specific management/treatment helpful and effective? Please specify per treatment.

_______________________________________ *(free text answer)*

1. If you selected any options in Question 39, which of the respiratory problems have the MOST impact on the quality of life in ADULTHOOD? Please specify.

_______________________________________ *(free text answer)*

1. GASTROINTESTINAL PROBLEMS
2. If the person with Malan syndrome currently has or has had gastrointestinal problems in ADULTHOOD, can you find them in the options below? (*Select all that apply*)
   1. Blood in stool
   2. Bloating
   3. Chronic/frequent diarrhea
   4. Constipation
   5. Trouble with swallowing (dysphagia)
   6. Gallstones
   7. Hemorrhoids (external, internal)
   8. Inflammatory Bowel Disease (i.e. Crohn’s, Ulcerative Colitis, etc)
   9. Irritable Bowel Syndrome
   10. Liver fibrosis
   11. Enlarged liver (hepatomegaly)
   12. Pancreatitis
   13. Recurrent/Cyclic vomiting
   14. Acid reflux/heartburn
   15. Small Bowel Obstruction
   16. Other
   17. Unsure
   18. No problems
3. If you answered ‘Other’ in Question 45, please specify.

_________________________________________ (*free text answer*)

1. If you chose one or more options in Question 45, please indicate the age when each gastrointestinal problem started/was diagnosed.
   __________________________________________ (*free text answer*)
2. If you chose one or more options in Question 45, what specific management/treatment was used? Please specify per problem.

__________________________________________ (*free text answer*)

1. If you filled in the previous question, did you find the specific management/treatment helpful and effective? Please specify per treatment.

__________________________________________ *(free text answer)*

1. If you answered ‘Constipation’ in Question 45, how frequently are stools coming in a week?

__________________________________________ (*free text answer*)

1. If you selected any options in Question 45, which of the gastrointestinal problems have the MOST impact on the quality of life in ADULTHOOD? Please specify.

_________________________________________ *(free text answer)*

1. SLEEP PROBLEMS
2. If the person with Malan syndrome currently has or has had sleep problems in ADULTHOOD, can you find them in the options below? (*select all that apply)*
3. Difficulty falling asleep
4. Difficulty staying asleep/awaking frequently during the night
5. Increased need for sleep
6. Falling asleep suddenly during the day (narcolepsy)
7. Breathing repeatedly stops and starts during sleep or the person snores loudly and feels tired even after a full night’s sleep (Sleep Apnea)
8. Unpleasant or uncomfortable sensations in the legs and an irresistible urge to move them. Symptoms commonly occur in the late afternoon or evening hours and are often most severe at night when a person is resting, such as sitting or lying in bed (Restless Legs Syndrome or Periodic Limb Movement Disorder).
9. Other
10. Unsure
11. No problems
12. If you answered ‘Other’ in Question 52, could you specify?

_______________________________ (*free text answer*)

1. If you answered ‘Increased need for sleep’ in Question 52, which one of the options below best describes the problem? *(select all that apply)*
   1. Increased nighttime sleeping
   2. Increased daytime sleeping
   3. Increased need to sleep immediately after lunch
   4. Mood change if not allowed to sleep
   5. Other
2. If you answered ‘Other’ in Question 54, please specify.

_______________________________ (*free text answer*)

1. If you chose one or more options in Question 52, please indicate the age when each sleep problem started/was diagnosed.

_____________________________________ *(free text answer)*

1. If you chose one or more options in Question 52, what specific management/treatment was used? Please specify per problem.

_____________________________________ (*free text answer*)

1. If you filled in the previous question, did you find the specific management/treatment helpful and effective? Please specify per treatment.

_______________________________________ *(free text answer)*

1. If you selected any options in Question 52, which of the sleep problems have the MOST impact on the quality of life in ADULTHOOD? Please specify.

_____________________________________ *(free text answer)*

1. VISION AND HEARING PROBLEMS
2. If the person with Malan syndrome currently has or has had vision problems in ADULTHOOD, can you find them in the options below? *(select all that apply)*
   1. Vision decline
   2. Optic atrophy (stable)
   3. Optic atrophy (progressive)
   4. Cataracts
   5. Macular degeneration
   6. Strabismus
   7. Nystagmus
   8. Other
   9. Unsure
   10. No problems
3. If you answered ‘Other’ in Question 60, please specify.
   ____________________________________ *(free text answer)*
4. If you chose one or more problems in Question 60, please indicate the age when each vision problem started or was diagnosed.
   ____________________________________ *(free text answer)*
5. Does the person with Malan syndrome use any assistive devices to help with vision? (for example: glasses, white cane, magnifiers, colored lenses etc)
   ____________________________________ *(free text answer)*
6. If you selected any options in Question 60, which of the vision problems have the MOST impact on the quality of life in ADULTHOOD? Please specify.
   ____________________________________ *(free text answer)*
7. If the person with Malan syndrome currently has or has had hearing problems in ADULTHOOD, can you find them in the options below? *(Select all that apply)*
   1. Hearing decline
   2. Conductive hearing loss
   3. Sensorineural hearing loss
   4. Hypersensitivity to noise
   5. Other
   6. Unsure
   7. No problems
8. If you answered ‘Other’ in Question 65, please specify.
   ____________________________________ *(free text answer)*
9. If you chose one or more problems in Question 65, please indicate the age when each hearing problem started or was diagnosed.
   _____________________________________ *(free text answer)*
10. Does the person with Malan syndrome wear hearing aids?
    1. Yes
    2. No
11. If you selected any options in Question 65, which of the hearing problems have the MOST impact on the quality of life in ADULTHOOD? Please specify.
    __________________________________ *(free text answer)*
12. BEHAVIOURAL OR PSYCHIATRIC PROBLEMS
13. If the person with Malan syndrome currently has or has had behavioral/psychiatric problems in ADULTHOOD, can you find them in the options below? (*select all that apply*)
14. Aggression toward others
15. Anxiety
16. Autistic behaviour
17. Depression
18. Difficulty maintaining attention, hyperactivity and impulsive behaviour, also known as Attention Deficit Hyperactivity Disorder (ADHD)
19. High levels of frustration
20. Limited interests and repetition (routine/words/order of doing things)
21. Loss of interest in things that used to interest them
22. Loss of skills that they used to be able to perform
23. Mood abnormalities or disorder/sudden mood changes
24. Recurring, unwanted thoughts, ideas or sensations (obsessions) that make them feel driven to do something repetitively (compulsions), also known as Obsessive Compulsive Disorder (OCD)
25. Self-injurious behavior/self-harm
26. Sudden onset of reduced activity
27. Other
28. Unsure
29. No problems
30. If you answered ‘Other’ in Question 70, please specify.

___________________________________ (*free text answer*)

1. If you answered ‘Loss of skills’ in Question 70, please list all of them.

___________________________________ *(free text answer)*

1. If you chose one or more options in Question 70, please indicate the age when each behavioural/psychiatric problem started.
   ____________________________________ (*free text answer*)
2. If you chose one or more options in Question 70, what specific management/treatment was used? Please specify per problem.
   _____________________________________ (*free text answer*)
3. Was a specific management/treatment more helpful and effective than others and if so, which one and for which behavioural problem?

______________________________________ (*free text answer*)

1. If you selected any options in Question 70, which of the behavioural/psychiatric problems have the MOST impact on the quality of life in ADULTHOOD? Please specify.

_______________________________________(*free text answer*)

1. OTHER PROBLEMS
2. Does the person with Malan syndrome have any of the following in ADULTHOOD? (*Select all that apply)*
3. Arthrosis
4. Arthritis
5. Cancer
6. Decreased mobility/any difficulties with moving around.
7. Diabetes
8. Excessive eating
9. High pain threshold
10. Incontinence
11. Joint dislocation
12. Joint replacement
13. Ligament tears/injury
14. Menopause
15. Memory loss
16. New contractures
17. Obesity
18. Prostate problems (prostate enlargement)
19. Skin issues (for example: eczema)
20. Tremors
21. Underweight
22. Other
23. No problems
24. If you answered ‘Other’ in Question 77, please specify.

_________________________________________ (*free text answer*)

1. If you chose one or more options in Question 77, please indicate the age when each problem started/was diagnosed.

____________________________________________ (*free text answer*)

1. If you chose one or more options in Question 77, what specific management/treatment was used? Please specify per problem.

__________________________________________ (*free text answer*)

1. If you filled in the previous question, did you find the specific management/treatment helpful/effective? Please specify per treatment.

________________________________________ *(free text answer)*

1. If you selected any options in Question 77, which of the problems have the MOST impact on the quality of life in ADULTHOOD? Please specify.

________________________________________ *(free text answer)*

1. SURGERY
2. Has the person with Malan syndrome had any surgery during ADULTHOOD? (Select *one)*
   1. Yes
   2. No
3. If you answered ‘Yes’ in Question 83, please list all surgeries that the person with Malan syndrome has had during ADULTHOOD.
   ___________________________________________ (*free text answer*)
4. If you answered ‘Yes’ in Question 83, did the surgery resolve the issue(s)? Please specify.

___________________________________________ (*free text answer*)

1. Do you have any other comments about surgery in adulthood?
    ___________________________________________ (*free text answer*)
2. MEDICATION
3. Does the person with Malan syndrome take medication in ADULTHOOD?
   1. Yes
   2. No
4. If you answered ‘Yes’ in Question 87, how is medication taken?
   1. Independently WITHOUT help of dosette box/pill organizer/special packaging by pharmacy
   2. Independently WITH help of dosette box/pill organizer/special packaging by pharmacy
   3. With help of caregiver(s)
5. Does the person with Malan syndrome experience difficulties with taking medication?
   a. Yes
   b. No
6. If you answered ‘Yes’ in Question 89, please explain in what way the person with Malan syndrome experiences difficulties with taking medication.
   ____________________________________________________ (*free text answer*)
7. EVERYDAY LIFE
8. Does the adult Malan syndrome person have a job? (select *one*)
   1. Yes
   2. No
9. If you answered ‘Yes’ in Question 91, could you specify what kind of job?

_______________________________________ (*free text answer*)

1. Does the person with Malan syndrome maintain social activities and/or partake in sports? (select *one*)
   1. Yes
   2. No
2. If you answered ‘Yes’ in Question 93, could you specify what kind of social activities or sports?

_____________________________________ (*free text answer*)

1. Does the person with Malan syndrome drive a car? (select *one*)
   1. Yes
   2. No
2. Is the person with Malan syndrome able to take public transportation? (select *one*)
   1. Yes, independently
   2. Yes, with assistance
   3. No
3. Is the person with Malan syndrome able to do grocery shopping? (select *one*)
   1. Yes, independently
   2. Yes, with assistance
   3. No
4. It is important to know if individuals with Malan syndrome are sexually active. Please consider answering the following question: Is the person with Malan syndrome sexually active (select *one*)
5. Yes
6. No
7. I do not know
8. Prefer not to answer
9. If you answered ‘Yes’ to Question 98, please specify. (*select all that apply)*
   1. Stimulation of one’s own genitalia (masturbation)
   2. With a partner
   3. Other
10. If you answered ‘Other’ in Question 99, please specify.

_____________________________________ (*free text answer*)

1. How does the person with Malan syndrome communicate in ADULTHOOD? (*Select all that apply).*
   1. Verbal/spoken language
   2. Written language
   3. Sign language
   4. Picture Exchange Communication System
   5. Speech generating device
   6. Other
2. If you answered ‘Other’ in Question 101, please specify.

______________________________________ *(free text answer)*

1. If you answered ‘Verbal/spoken language’ in Question 101, what is their level of speech?
   1. 5+ word sentences
   2. 2 to 3 word combinations
   3. Single words
   4. Sounds
2. If you answered ‘Verbal/spoken language’ in Question 101, has their level of speech changed from childhood to adulthood?
   1. Yes, improved
   2. Yes, declined
   3. No changes
3. If you answered ‘Yes’ in Question 104, around what age did their level of speech change?

________________________ *(free text answer)*

1. Are there any other problems in everyday life that you/the person with Malan syndrome would like us to know about?

______________________________________________ (*free text answer*)

1. MEDICAL FOLLOW-UP
2. Who is monitoring the medical issues of the person with Malan syndrome in ADULTHOOD? (*select all that apply*)
3. The general practitioner
4. A specialist in the hospital
5. A multidisciplinary center with involvement of multiple specialists
6. Physician for people with intellectual disabilities
7. Nurse
8. Physiotherapist
9. Occupational therapist
10. Dentist and/or orthodontist
11. Dietitian
12. Other
13. No doctor is involved in adult life
14. If you answered ‘Other’ in Question 107, please specify.

_______________________________________ (*free text answer*)

1. If you answered ‘A specialist in the hospital’ in Question 107, could you specify which specialty or specialties?

_______________________________________ (*free text answer*)

1. If you answered ‘A multidisciplinary center’ in Question 107, could you specify which center and country?

_______________________________________ (*free text answer*)

1. How often does the person with Malan syndrome have health checks?
2. Once a year
3. Twice a year
4. Once every 3 years
5. Once every 5 years
6. Only if there are specific concerns.
7. Other
8. If you answered ‘Other’ in Question 111, could you specify?

_______________________________________ (*free text answer*)

1. Who is monitoring the mental health issues of the person with Malan syndrome in ADULTHOOD? *(Select all that apply)*
   1. Psychologist
   2. Behavioural specialist
   3. Psychiatrist
   4. Physician for people with intellectual disabilities
   5. Other
   6. None
2. If you answered ‘Other’ in Question 113, could you specify?
   _________________________________________ *(free text answer)*
3. Any final comments?

_________________________________________ (*free text answer*)
