# Supplementary material for: Natural history in Malan syndrome: survey of 28 adults and literature review
Source: Orphanet J Rare Dis. 2024 Jul 29;19:282. doi: 10.1186/s13023-024-03288-6 (PMC11288048; doi:10.1186/s13023-024-03288-6)
Supplement: Supplementary file 2 — Additional file 2. Supplemental material: table 1 literature review of adults with Malan syndrome. [file 13023_2024_3288_MOESM2_ESM.docx]

**Additional file 2 - Natural history in Malan syndrome: survey of 28 adults and literature review**

**Supplementary Table 1. Overview of characteristics of adults with Malan syndrome reported in literature.**

| **Study** |  | **Malan et al. 2010** | **Yoneda et al. 2012** | **Oshima et al. 2017** | **Priolo et al. 2018** | | | | | | | | | **Bellucco et al. 2019** | **Hancarova et al. 2019** | **Sihombing et al. 2020** | | | **Macchiaiolo et al. 2022** | | **Total**  **N = 19** |
| --- | --- | --- | --- | --- | --- | --- | --- | --- | --- | --- | --- | --- | --- | --- | --- | --- | --- | --- | --- | --- | --- |
| **Age (in years)** |  | 27 | 20 | 38 | 20 | 21^a^ | 21^b^ | 21^c^ | 22 | 24 | 31 | 42^d^ | 42^e^ | 19 | 23 | 22^f^ | 22^g^ | 26 | 18 | 25 | Median: 22 |
| **Gender** |  | F | M | M | F | M | M | F | M | M | F | M | M | M | M | F | F | F | F | F | 9F/10M |
| **Genotype** | NFIX variants | c.568C>T | c.362G>C | c.290_291insA | c.59T>C | c.779C>G | c.1117del | Deletion of complete NFIX | c.759C>G | c.325G>T | c.154_155insT | c.113G>T | c.383G>A | 990 kb deletion in 19p13.2p13.12 | c.346C>T | c.694C>T | c.694C>T | c.694C>T | c.1021del | c.198dup |  |
|  | Amino acid change | p.(Gln190*) | p.(Arg121Pro) | p.(Asp90Glufs829) | p.(Leu20Pro) | p.(Thr260Ser) | p.(ser373Profs*28) |  | p.(Tyr253*) | p.(Asp109Tyr) | p.(Glu52Valfs*67) | p.(Arg38Leu) | p.(Arg128Gln) |  | p.(Arg116Trp) | p.(Gln232*) | p.(Gln232*) | p.(Gln232*) | p.His341ThrsTer52 | p.Glu67ArgfsTer60 |  |
| **Newborn measurements** | Birth weight >2SDS | - | - | - | - | - | - | - | - | - | - | ? | - | - | - | ? | ? | - | - | - | 0/16 |
|  | Birth length >2SDS | - | - | - | - | - | - | - | + | - | + | ? | - | - | - | ? | ? | - | - | - | 2/16 |
|  | OFC >2SDS | + | - | + | ? | - | + | + | + | - | + | ? | + | + | ? | ? | ? | - | + | - | 9/14 |
| **Postnatal growth** | Weight >2SDS | ? | - | - | ? | ? | ? | ? | ? | ? | ? | ? | ? | - | ? | ? | ? | ? | ? | ? | 0/3 |
|  | Height >2SDS | + | + | - | + | - | - | - | + | + | - | + | + | - | ? | + | + | + | + | - | 11/18 |
|  | OFC >2SDS | + | ? | + | + | + | + | - | + | + | - | + | + | - | ? | + | + | + | + | + | 14/17 |
| **Craniofacial** | Dental overcrowding | ? | ? | ? | ? | ? | ? | ? | + | ? | ? | ? | ? | ? | ? | ? | ? | ? | + | + | 3/3 |
|  | Deep set eyes | + | ? | - | + | + | + | + | + | - | - | + | + | + | + | + | + | + | - | - | 13/18 |
|  | Down slanting palpebral fissures | + | + | + | + | - | - | - | + | + | + | + | + | + | + | + | + | + | - | + | 15/19 |
|  | Everted lower lip | + | - | ? | + | + | - | - | - | - | + | + | - | - | + | - | - | - | + | + | 8/18 |
|  | High and prominent forehead | + | + | + | + | + | + | + | + | + | + | + | + | + | + | + | + | + | + | + | 19/19 |
|  | Prominent chin | - | + | + | + | - | + | + | + | + | + | + | + | - | + | + | + | + | + | + | 16/19 |
|  | Thin upper lip | - | - | ? | - | - | - | + | + | - | - | - | + | - | - | - | - | - | - | + | 4/18 |
|  | Small mouth | + | - | ? | + | + | - | - | + | + | + | + | + | - | - | - | - | - | - | + | 9/18 |
|  | Long, narrow, and triangular face | + | + | ? | + | + | + | + | + | + | + | + | + | + | + | + | + | + | + | + | 18/18 |
|  | Depressed nasal bridge | ? | + | ? | - | - | + | - | - | - | - | - | - | - | - | + | + | - | - | - | 4/17 |
|  | Short nose (SO)/ anteverted nares (AN) | - | - | ? | AN | SO, AN | SO | - | - | SO | SO | - | - | AN | - | - | SO | - | - | SO, AN | 4AN/6SO |
| **Developmental** | Intellectual disability | + | + | + | + | + | + | + | + | + | + | + | + | + | + | + | + | + | + | + | 19/19 |
| **Psychobehavioral** | Anxiety | + | ? | ? | ? | ? | ? | ? | ? | ? | ? | - | ? | + | - | ? | ? | ? | + | + | 4/6 |
|  | Autistiform traits | + | - | ? | - | + | - | - | + | + | - | - | + | - | + | - | - | - | + | + | 8/18 |
|  | Attention Deficit Hyperactivity Disorder (ADHD) | ? | ? | ? | ? | ? | ? | ? | ? | ? | ? | - | ? | - | ? | ? | ? | ? | - | - | 0/4 |
|  | Other psychobehavioral features | + | - | ? | + | + | - | + | + | + | + | - | + | - | ? | ? | ? | ? | ? | ? | 8/12 |
| **Neurological** | Seizures/EEG anomalies | ? | ? | + | - | - | - | - | + | - | - | + | - | + | ? | - | + | - | + | + | 7/16 |
|  | Brain MRI anomalies | + | ? | ? | + | - | + | - | + | - | - | - | ? | + | + | ? | ? | ? | + | + | 8/13 |
|  | Hypotonia | - | + | ? | + | ? | + | + | + | + | - | - | + | + | + | + | + | + | + | - | 13/17 |
| **Musculoskeletal** | Slender habitus | + | + | + | - | + | - | + | + | - | + | - | + | + | ? | + | + | + | + | + | 14/18 |
|  | Advanced bone age | + | ? | + | - | + | ? | - | + | + | + | ? | + | ? | ? | ? | ? | ? | ? | ? | 7/9 |
|  | Scoliosis | + | - | + | - | + | - | + | + | - | - | - | - | + | + | + | + | + | + | + | 12/19 |
|  | Kyphosis (K)/lordosis (L) | - | - | K | - | K | - | K | K | - | - | - | - | - | K | K | K | K | - | L | 8K/1L |
|  | Pectus excavatum (PE)/pectus carinatum (PC) | - | - | PE | - | + | - | + | + | - | - | - | - | PC | PE | - | ? | + | - | + | 8/18 |
|  | Long hands/fingers | - | ? | + | - | - | - | + | + | + | ? | ? | + | + | + | + | - | + | + | + | 11/16 |
|  | Joint hyperlaxity | ? | ? | ? | + | + | - | - | - | - | + | - | - | ? | ? | - | - | - | + | - | 4/14 |
|  | Bone fractures | ? | - | ? | ? | ? | ? | ? | ? | ? | ? | ? | ? | ? | ? | ? | ? | ? | + | + | 2/3 |
|  | Pes planus | ? | ? | ? | ? | ? | ? | ? | ? | ? | ? | ? | ? | ? | ? | ? | ? | ? | + | + | 2/2 |
| **Vision/eyes** | Refractive disorders (M, H, A) | - | M-, H-, A? | ? | - | A | - | - | M, H, A | A | H | - | M | ? | H | - | - | - | M, A | M, A | 5A, 3H, 4M |
|  | Blue sclerae | - | - | ? | - | - | - | - | - | - | + | - | - | - | - | ? | ? | ? | + | + | 3/15 |
|  | Strabismus | + | - | ? | + | + | - | + | + | - | - | - | - | + | - | + | + | + | + | - | 10/18 |
|  | Nystagmus | + | - | ? | - | - | - | - | + | - | - | - | - | ? | ? | ? | ? | ? | - | - | 2/13 |
|  | Cataract | - | ? | ? | - | - | - | - | - | - | - | - | - | ? | - | - | - | - | - | - | 0/16 |
| **Hearing/ears** | Hearing loss | ? | ? | ? | ? | ? | ? | ? | ? | ? | ? | ? | ? | ? | ? | ? | ? | ? | - | - | 0/2 |
|  | Noise hypersensitivity | ? | ? | ? | ? | ? | ? | ? | ? | ? | ? | ? | ? | ? | ? | ? | ? | ? | + | + | 2/2 |
|  | Recurrent otitis media | ? | + | ? | ? | ? | ? | ? | ? | ? | ? | ? | ? | ? | ? | ? | ? | ? | ? | ? | 1/1 |
| **Gastrointestinal** | Constipation | ? | ? | ? | ? | ? | ? | ? | ? | ? | ? | ? | ? | ? | ? | ? | ? | ? | - | - | 0/2 |
| **Cardiovascular** | Aortic root dilation | ? | ? | - | ? | ? | ? | ? | ? | ? | ? | ? | ? | ? | ? | ? | ? | ? | - | - | 0/3 |
|  | Pulmonary artery dilation | ? | ? | - | ? | ? | + | ? | ? | ? | ? | ? | ? | ? | ? | ? | ? | ? | ? | ? | 1/2 |
|  | Mitral valve regurgitation | ? | ? | - | ? | ? | ? | ? | ? | ? | ? | ? | ? | ? | ? | ? | ? | ? | + | - | 1/3 |
|  | Other cardiovascular anomalies | ? | - | Hypertension, thoracic aortic aneurysm, mild aortic regurgitation, and thoracic aortic dissection | ? | ? | ? | ? | ? | ? | ? | ? | ? | ? | ? | ? | ? | ? | ? | ? | 1/2 |

**Legend.** M: male, F: female, + present, - absent, ? unknown, OFC: occipital frontal circumference, H: hypermetropia, M: myopia, A: astigmatism, EEG: electroencephalogram

^a^Patient ID: 38

^b^Patient ID: 42

^c^Patient ID: 45

^d^Patient ID: 6

^e^Patient ID: 27

^f^Twin sister II:3

^g^Twin sister II:4
