# Supplementary material for: Natural history in Malan syndrome: survey of 28 adults and literature review
Source: Orphanet J Rare Dis. 2024 Jul 29;19:282. doi: 10.1186/s13023-024-03288-6 (PMC11288048; doi:10.1186/s13023-024-03288-6)
Supplement: Supplementary file 3 — Additional file 3. Supplemental material: Tables 2-5 and Figures 1-2. [file 13023_2024_3288_MOESM3_ESM.docx]

**Additional file 3 - Natural history in Malan syndrome: survey of 28 adults and literature review**

***Supplemental tables***

**Supplementary Table 2. Genotypical data of adult individuals with Malan syndrome in present cohort.**

| **Participant ID** | **Gene** | **DNA** | **Amino acid change** | **Type of mutation** | **Microdeletion** |
| --- | --- | --- | --- | --- | --- |
| 5 | NFIX |  |  |  | 1Mb deletion 19p13.2 |
| 6 | NFIX | c.449T>C | p.Leu150Pro |  |  |
| 8 | NFIX | c.113G>T | p.Arg38Leu | Missense (NFIX) |  |
| 9* | NFIX |  |  |  |  |
| 10 | NFIX | c.298_299delinsC | p.(Val100Argfs*2) | Frameshift |  |
| 11 | NFIX | c.565_566insA | p.Tyr189Ter | Nonsense |  |
| 12 | NFIX | c.116T>C | p.Phe39Ser | Missense |  |
| 13 | NFIX | c.371G> | p.(Arg124Gln) | Missense |  |
| 14 | NFIX, CACNA1A |  |  |  | Deletion of both genes |
| 15 | NFIX | c.335 T>A | p.Val112Glu | Missense |  |
| 16 | NFIX | c.264del | p.(Lys89Argfs*13) | Frameshift |  |
| 17 | CACNA1A, NFIX, CC2D1A |  |  |  | 1.3 Mb deletion 19p13.2p13.12 |
| 18* | NFIX | c.191delA | p.Lys64SerfsTer30 | Frameshift |  |
| 19* | NFIX | c.1021del | p.His341ThrsTer52 | In frame deletion |  |
| 20* | NFIX | c.157_177del | p.Glu53_Glu59del | In frame deletion |  |
| 21* | NFIX |  |  |  | 687-793 Kb deletion 19p13.2 |
| 22* | NFIX | c.198dup | p.Glu67ArgfsTer60 | In frame duplication |  |
| 23 | NFIX |  |  |  | 1.2 Mb deletion 19p13.2 |
| 25 | NFIX |  |  |  | 349.781 kb deletion 19p13.2 |
| 26^ | NFIX |  |  |  |  |
| 27 | NFIX | c.740C>G | p.Ser247Ter | Nonsense |  |
| 28 | NFIX | c.759C>G | p.Tyr253Ter | Nonsense |  |
| 29 | NFIX | c.842+1G>A |  | Splicing |  |
| 30* | NFIX |  |  |  |  |
| 31 | NFIX | c.180_187dupGCTGGGCG | p.Glu63GlyfsX34 | Frameshift |  |
| 32^ | NFIX |  |  |  |  |
| 33 | NFIX |  |  |  | Heterozygous deletion of exons 9 and 10 in NFIX gene |
| 34 | NFIX | c.406C>T | p.Arg136Trp | Missense |  |

**Legend.** * No genetic report, genetic diagnosis confirmed via physician. ^ Genetic report not available: diagnosis via 100,000 genome project. **Supplementary Table 3. Minor neurological, musculoskeletal, vision and psychobehavioral signs.**

|  | **Sign** | **Number of individuals (%)** |
| --- | --- | --- |
| **Neurological** | Chiari malformation | 1 (3.6%) |
|  | Hemiplegic migraine | 2 (7.1%) |
| **Musculoskeletal** | Pelvic bone abnormality | 2 (7.1%) |
|  | Tibial bowing | 2 (7.1%) |
|  | Flat feet | 2 (7.1%) |
|  | Trouble with breathing | 2 (7.1%) |
|  | Abnormal sensations in muscles | 2 (7.1%) |
| **Vision** | Chiasma and optic nerve hypoplasia | 1 (3.6%) |
|  | Cortical visual impairment | 1 (3.6%) |
|  | Color blindness | 1 (3.6%) |
|  | Photophobia | 1 (3.6%) |
|  | Impaired visuospatial perception | 2 (7.1%) |
|  | Optic disc pallor | 1 (3.6%) |
|  | Dissociated vertical deviation | 1 (3.6%) |
| **Psychobehavioral** | Sudden onset of reduced activity | 2 (7.1%) |
|  | Panic attacks | 1 (3.6%) |
|  | Loss of skills | 2 (7.1%) |

**Supplementary Table 4. Cardiovascular, respiratory, gastrointestinal, sleep, hearing and other reported signs.**

|  | **Sign** | **Number of individuals (%)** |
| --- | --- | --- |
| **Cardiovascular** | Dilated left ventricle | 1 (3.6%) |
|  | Tachycardia | 1 (3.6%) |
|  | Hypertension | 1 (3.6%) |
|  | Valve defect | 3 (10.7%) |
|  | Dilated aorta | 3 (10.7%) |
|  | Heart murmur | 3 (10.7%) |
| **Respiratory** | Bronchiectasis | 1 (3.6%) |
|  | Adenoids hypertrophy | 1 (3.6%) |
|  | Asthma | 2 (7.1%) |
|  | Full/partial lung collapse | 1 (3.6%) |
|  | Recurrent pneumonia | 2 (7.1%) |
|  | Reduced lung capacity | 2 (7.1%) |
|  | Sarcoidosis | 1 (3.6%) |
|  | Acute bronchitis | 3 (10.7%) |
| **Gastrointestinal** | Dysphagia | 1 (3.6%) |
|  | Lactose intolerance | 1 (3.6%) |
|  | Inflammatory Bowel Disease | 1 (3.6%) |
|  | Blood in stool | 3 (10.7%) |
|  | Chronic/frequent diarrhea | 2 (7.1%) |
|  | Recurrent vomiting | 3 (10.7%) |
|  | Bloating | 4 (14.3%) |
|  | Acid reflux | 3 (10.7%) |
|  | Hemorrhoids | 7 (25%) |
|  | Constipation | 15 (53.6%) |
| **Sleep** | Restless Legs Syndrome | 2 (7.1%) |
|  | Parasomnia | 1 (3.6%) |
|  | Difficulty falling asleep | 4 (14.3%) |
|  | Sleep apnea | 6 (21.4%) |
|  | Difficulty staying asleep/awaking frequently during the night | 9 (32.1%) |
|  | Increased need for sleep | 9 (32.1%) |
| **Hearing** | Hearing decline | 2 (7.1%) |
|  | Conductive hearing loss | 2 (7.1%) |
|  | Cerumen impaction | 1 (3.6%) |
|  | Recurrent ear infections | 1 (3.6%) |
|  | Sensorineural hearing loss | 2 (7.1%) |
|  | Hypersensitivity to noise | 13 (46.4%) |
| **Other** | Arthritis | 2 (7.1%) |
|  | Decreased mobility | 4 (14.3%) |
|  | Diabetes | 1 (3.6%) |
|  | Excessive eating | 6 (21.4%) |
|  | High pain threshold | 13 (46.4%) |
|  | Incontinence | 7 (25%) |
|  | Joint hypermobility | 6 (21.4%) |
|  | Joint replacement | 1 (3.6%) |
|  | Ligament tears/injury | 1 (3.6%) |
|  | Memory loss | 1 (3.6%) |
|  | Contractures | 3 (10.7%) |
|  | Skin issues | 9 (32.1%) |
|  | Underweight | 5 (17.9%) |
|  | Hypersensitivity to smell | 1 (3.6%) |
|  | Joint hypomobility, elbow and shoulder joints less range of motion, resisting full extension of elbows | 1 (3.6%) |
|  | Obesity | 3 (10.7%) |

**Supplementary Table 5. Detailed information on past and current medication of all adult individuals with MALNS.**

| **Category** | **Name** | **Medical issue(s)** | **With or without benefit (number of individuals)** |
| --- | --- | --- | --- |
| Antiepileptics | Levetiracetam, Lacosamide, Brivaracetam, Gabapentin, Lamotrigine, Oxcarbazepine, Divalproex sodium, Clonazepam, Zonisamide | Seizures, anxiety | Levetiracetam: + (N=3)  Brivaracetam: + (N=1)  Gabapentin: + (N=2)  Lamotrigine: + (N=2), - (N=1)  Oxcarbazepine: + (N=1)  Divalproex sodium: + (N=1), - (N=1)  Clonazepam: + (N=3), - (N=1) |
| Benzodiazepines | Alprazolam | Panic attacks | N/a |
|  | Clotiazepam | Self-injurious behavior, rage attacks | N/a |
|  | Clobazam | Seizures | + (N=2) |
|  | Lorazepam, Diazepam | Anxiety | Lorazepam: - (N=1)  Diazepam: - (N=1) |
| Carbonic anhydrase inhibitors | Diamox | Dizziness, vertigo, recurrent vomiting | N/a |
| Antipsychotics | Risperidone, Aripiprazole, Quetiapine | Anxiety | Risperidone: + (N=1)  Aripiprazole: - (N=1)  Quetiapine: + (N=1) |
|  | Olanzapine | Aggression | N/a |
| SSRIs | Sertraline | Anxiety, depression | + (N=1), - (N=1) |
|  | Fluoxetine | Anxiety, mood abnormalities | + (N=1), - (N=1) |
|  | Escitalopram, Citalopram, Fluvoxamine, Paroxetine | Anxiety | Escitalopram: + (N=1), - (N=1)  Citalopram: + (N=1)  Paroxetine: + (N=1)  Fluvoxamine: - (N=1) |
| SNRIs | Venlafaxine | Anxiety | - (N=1) |
| TCAs | Mirtazapine | Anxiety | N/a |
| Psychostimulants | Methylphenidate (Ritalin) | High stress situations | N/a |
| Laxatives | Metamucil, Macrogol, Miralax, Restoralax | Constipation | + (N=9) |
| Proton pump inhibitors | Omeprazole | Acid reflux | + (N=1) |
| Serotonin 5-HT_3_ receptor antagonists | Ondansetron | Recurrent vomiting | + (N=1) |
| Other | Buspiron | Anxiety | + (N=1) |
| Antihypertensives | Clonidine, Guanfacine | Behavior | - (N=1) |
|  | Atenolol | Hypertension | + (N=1) |

**Legend.** SSRIs: selective serotonin reuptake inhibitors, SNRIs: serotonin and norepinephrine reuptake inhibitors, TCAs: tricyclic antidepressants, N/a: no answer, +: with benefit, -: without benefit.

***Supplemental figures***

**Supplementary Figure 1. Most common phenotypical characteristics of adults with MALNS reported in literature compared to current cohort.**

**Legend.** SDS: standard deviation score

**Supplementary Figure 2.** **Age distribution among 28 adult individuals.**
